# Identification of Reference Proteins for Western Blot Analyses in Mouse Model Systems of 2,3,7,8-Tetrachlorodibenzo-P-Dioxin (TCDD) Toxicity

**DOI:** 10.1371/journal.pone.0110730

**Published:** 2014-10-17

**Authors:** Stephenie D. Prokopec, John D. Watson, Raimo Pohjanvirta, Paul C. Boutros

**Affiliations:** 1 Informatics and Bio-computing Program, Ontario Institute for Cancer Research, Toronto, Ontario, Canada; 2 Laboratory of Toxicology, National Institute for Health and Welfare, Kuopio, Finland; 3 Department of Food Hygiene and Environmental Health, University of Helsinki, Helsinki, Finland; 4 Department of Medical Biophysics, University of Toronto, Toronto, Ontario, Canada; 5 Department of Pharmacology & Toxicology, University of Toronto, Toronto, Ontario, Canada; Nanjing Medical University, China

## Abstract

Western blotting is a well-established, inexpensive and accurate way of measuring protein content. Because of technical variation between wells, normalization is required for valid interpretation of results across multiple samples. Typically this involves the use of one or more endogenous controls to adjust the measured levels of experimental molecules. Although some endogenous controls are widely used, validation is required for each experimental system. This is critical when studying transcriptional-modulators, such as toxicants like 2,3,7,8-tetrachlorodibenzo-p-dioxin (TCDD).To address this issue, we examined hepatic tissue from 192 mice representing 47 unique combinations of strain, sex, *Ahr*-genotype, TCDD dose and treatment time. We examined 7 candidate reference proteins in each animal and assessed consistency of protein abundance through: 1) TCDD-induced fold-difference in protein content from basal levels, 2) inter- and intra- animal stability, and 3) the ability of each candidate to reduce instability of the other candidates. Univariate analyses identified HPRT as the most stable protein. Multivariate analysis indicated that stability generally increased with the number of proteins used, but gains from using >3 proteins were small. Lastly, by comparing these new data to our previous studies of mRNA controls on the same animals, we were able to show that the ideal mRNA and protein control-genes are distinct, and use of only 2–3 proteins provides strong stability, unlike in mRNA studies in the same cohort, where larger control-gene batteries were needed.

## Introduction

2,3,7,8-tetrachlorodibenzo-*p*-dioxin (TCDD) is a member of a class of environmental contaminants, known as dioxins, and is primarily produced through industrial processes including incineration and manufacture of herbicides and pesticides [1], [2] as well as electronics recycling [3]. Exposure to TCDD evokes a wide range of toxicities in laboratory animals, including wasting syndrome and death [4]. In humans, short-term exposure to high levels of TCDD often presents as liver damage and chloracne, while low-dose long-term exposure has been linked to immune deficiency [5], diabetes [6], and various cancer types [2], [7].

TCDD is an exogenous ligand for the aryl hydrocarbon receptor (AHR) [8]. Upon cell entry, TCDD binds cytoplasmic AHR, leading to the formation of a ligand-receptor complex which translocates into the nucleus, dimerizes with the AHR nuclear translocator (ARNT) and binds to DNA to regulate transcription of target genes [9]. Previous studies have shown that TCDD exposure results in the dysregulation of hundreds of genes in numerous models [10], [11], [12], [13], [14]. While specific changes to the transcriptome resulting from TCDD-mediated regulation have been identified across a wide range of experimental models, downstream effects on the proteome which may prove causative of toxicities, remain unclear. Complete examination of various –omics data will be required to identify the specific molecules responsible for the severe toxic effects induced by TCDD.

Animal models have been, and will continue to be, crucial to understanding the mechanisms described above [15]. In particular, the varying sensitivities to TCDD of different species and strains of rodent greatly contribute to our understanding of TCDD-mediated toxicities. For example, the Long-Evans rat strain (*Turku/AB*; L-E) displays a very low tolerance for TCDD (LD_50_ = 10 µg/kg) while the Han/Wistar rat (*Kuopio*; H/W) is resistant to TCDD-induced lethality (LD_50_>9600 µg/kg) [16]. This difference in sensitivity is caused by a point mutation in the H/W *Ahr*, resulting in expression of multiple isoforms of the AHR [17], leading to differential regulation of a subset of genes in H/W animals [18]. These differentially abundant transcripts, and any ensuing changes to the proteome, may lead to strain-specific TCDD toxicities. Similarly, in mice, both the C57BL/6 and DBA/2 strains exhibit TCDD-mediated toxic effects, however DBA/2 mice are much more resistant (approximately 10 to 20 times) than the C57BL/6 strain [19]. This resistance is caused by a point mutation within the ligand binding domain of the *Ahr* in the DBA/2 mice [20]. TCDD-toxicity also varies between male and female animals within a species. Female rats are more sensitive to TCDD-lethality than male rats, while in mice this relationship is reversed [21].

Analysis of protein content is the general end-point for many biological experiments. While mass spectrophotometry is a highly sensitive and specific technique, both the data generation and analysis steps are highly complex [22]. As such, western blot has become the standard method of use, as it allows for the sensitive and specific detection of target proteins with accurate relative quantitation of protein content in a relatively simple and inexpensive manner [23]. However, as in transcriptomic studies, accurate assessment of protein abundance by western blot requires thorough normalization of the data prior to the interpretation of results. This normalization typically involves the use of total protein or one or more endogenous loading controls in order to account for technical variability and to determine relative target abundance, thereby allowing multiple samples to be compared. While measurement of total protein is a relatively simple approach, it leads to complications downstream [24]. Specifically, coomassie stained gels cannot be transferred to membrane for subsequent analysis and thereby requires the assumption that simultaneously run gels are loaded with identical amounts of protein [25]. The use of endogenous controls bypasses the need for additional steps, thereby reducing the number of gels and amount of sample used. Ideal endogenous control proteins maintain consistent levels of abundance regardless of environmental conditions, and thus often perform functions essential for cell survival [26]. Glyceraldehyde-3-phosphate dehydrogenase (GAPDH) and beta-actin (ACTB) have frequently been used as reference genes for both mRNA expression measured by qPCR [26], [27] and western blot analyses of protein content [28]. However, studies have shown that the stability of these widely used reference genes is not always consistent under different experimental conditions [29], [30]. Factors such as tissue-type [30], organism (between and within species) [31], experimental manipulation [32] and even reagents used [33] can affect the abundance of candidate reference molecules. For these reasons, it is essential that endogenous reference proteins be thoroughly evaluated prior to experimental use.

Investigations into TCDD-induced proteomic changes are necessary to further our understanding of dioxin toxicity. Before these studies can proceed, candidate reference proteins must be carefully validated for use in western blot within the model systems used. Several reference genes have been previously validated for use in transcriptomic studies in rat [34] and mouse models [31] of TCDD toxicity. Currently, reference proteins for use in proteomic studies within these animal models have yet to undergo thorough validation. Since the transcriptomic responses differ dramatically across animal models [14], [35], it is unclear whether these validated transcriptomic reference genes will translate to proteomic studies in either species. While it is not necessary to use the same controls for assessments of both gene and protein abundance, it is generally accepted that stably expressed genes may result in consistent abundance of protein [36], [37]. We therefore chose to examine those genes previously identified as suitable references for transcriptomic studies of TCDD-toxicity [31], in addition to ACTB, to determine their validity for proteomic studies. Seven candidate proteins (*i.e.* ACTB, EEF1A1, GAPDH, HPRT, PGK1, PPIA and SDHA) were tested in hepatic tissue from multiple mouse models of TCDD-toxicity. This allows us to experimentally verify the idea that similar controls can be used at the RNA and protein levels, which would reduce the workload inherent in establishing controls.

## Methods

### Ethics Statement

All study plans were approved by the Finnish National Animal Experiment Board (Eläinkoelautakunta, ELLA; permit code: ESLH-2008-07223/Ym-23).

### Animal Handling

Animal models and handling have been described previously [31]. Briefly, mouse colonies were maintained at the National Public Health Institute (today National Institute for Health and Welfare), Division of Environmental Health, Kuopio, Finland. Male and female C57BL/6 wild-type mice [21], male transgenic mice [38] and male DBA/2J mice [21] were studied. Wild-type animals were 12–15 weeks old and transgenic mice ranged up to 23 weeks. Animals were housed singly to avoid aggressive social behaviour, with environmental conditions maintained at 21±1°C with a relative humidity of 50 ± 10% on a 12 hour light cycle (12 hours of light followed by 12 hours of dark). Housing consisted of suspended, wire-mesh stainless-steel cages or Makrolon cages with aspen chip bedding (Tapvei Oy, Kaavi, Finland) and animals were provided with Altromin 1314 pellet feed (Altromin Spezialfutter GmbH & Co. KG, Lage, Germany) and water available *ad libitum*. The microbiological status of the animal facilities was regularly monitored in compliance with the recommendations of the Federation of European Laboratory Animal Science (FELASA), but individual mice were not tested in this regard. All experimental animals were drug and test naïve. Initial body weights for each animal are provided in Table S6.

Animals were stratified according to age such that groups contained a similar age-range, followed by randomization into experimental groups. Mice were treated in a group-wise manner, starting with the control in order to minimize the chance of human error. In most cases, the administration for a group was accomplished within an hour. Mice were treated with TCDD or corn oil vehicle alone and assessed following both timecourse and dose-response studies as described previously [31]. A total of 192 mice were used distributed across 47 separate experimental conditions (Table 1, Figure S1). TCDD was dissolved in corn oil and administered by oral gavage (10 mL/kg). Mice treated with corn oil alone acted as controls in each experiment.

**Table 1:** Experimental Design.

Study	Strain	Sex	Genotype	Treatment (TCDD µg/kg)	Time of tissue harvest (hours)	Number of animals
1	C57BL/6	Male	WT	0, 500	6	4, 5
	C57BL/6	Female	WT	0, 500	6	4, 5
2	C57BL/6	Male	rWT	0, 5, 500	19	4, 4, 4
	DBA/2J	Male	Ala375Val	0, 5, 500	19	4, 4, 4
3	C57BL/6	Male	WT	0, 500	24	4, 5
	C57BL/6	Female	WT	0, 500	24	3, 5
4	C57BL/6	Male	WT	0, 500	72	4, 5
	C57BL/6	Female	WT	0, 500	72	4, 5
5	C57BL/6	Male	WT	0, 500	144	3, 4
	C57BL/6	Female	WT	0, 500	144	3, 5
6	C57BL/6	Male	WT	0, 125, 250, 500, 1000	96	4, 4, 4, 4, 4
7	C57BL/6	Male	DEL	0, 125, 250, 500, 1000	96	5, 4, 3, 3, 4
8	C57BL/6	Male	INS	0, 125, 250, 500, 1000	96	5, 4, 4, 4, 5
9	C57BL/6	Male	rWT	0, 125, 250, 500, 1000	96	5, 3, 1, 4, 3
10	C57BL/6	Female	WT	0, 125, 250, 500, 1000	96	5, 5, 4, 4, 5

Animals analyzed (n = 192) varied in strain, sex, *Ahr*-allele, TCDD-treatment and time-point at which tissue was collected.

Briefly, animals in the timecourse study were treated with a single dose of TCDD (500 µg/kg) or corn oil alone at time zero, followed by euthanasia at different time points (animals with tissue collected at the 19 hour time point received either 0, 5 or 500 µg/kg TCDD). Animals in the dose-response study received a single dose of 0, 125, 250, 500 or 1000 µg/kg TCDD followed by euthanasia 96 hours post-treatment. Although some of these doses were above the LD_50_ level of the exposed animals, the exposure time was in all cases maximally about 50% of the shortest time-to-death for these strains and genetic models as recorded in previous studies [21], [33], and no mortality was therefore expected. However, all animals were carefully observed at least twice daily throughout the experimental period and, should signs consistent with severe suffering have been detected, those animals would have been euthanized immediately, as per the approved animal study plans.

Mouse livers were excised and snap-frozen in liquid nitrogen following euthanasia by carbon dioxide exposure. Tissue was shipped on dry ice to the analytical laboratory and stored at −80°C or colder. All animal handling and reporting comply with ARRIVE guidelines [39].

### Western analysis

Protein levels for candidate genes were determined by quantitative western blot. Each experiment was assessed on a single western blot to ensure identical analysis conditions between treated and control animals. Total protein was isolated from mouse liver using Tissue Extraction Reagent I (Life Technologies, Burlington, ON) supplemented with cOmplete protease inhibitor cocktail (Roche, Laval, QC). Protein extract, diluted 1/10 and 1/20 with 1XPBS, was quantified by Bradford assay and diluted to a final concentration of 10 µg/µL. A total of 65 µg protein [40], [41] was loaded into each well of a Novex 4–12% Bis-Tris midi-gel system to ensure sufficient material would be available for the detection of low abundance targets [42]. Prepared gels were then electrophoresed for 40 minutes at 200V with MES running buffer (Life Technologies). Protein was transferred to PVDF membrane with the iBlot system using program P0 for 7 minutes (Life Technologies). The Colloidal Blue Staining Kit (Life Technologies) was used to observe total protein before and after electrophoresis and Ponceau staining was performed on the transferred membrane to ensure sufficient protein transfer (Figure S4). While there is some variation between samples, protein transfer appears consistent. Primary antibodies were purchased from Santa Cruz (Santa Cruz Biotechnology Inc., Dallas, TX) or Abcam (Abcam Inc., Toronto, ON) and were diluted at the recommended concentrations in Li-Cor blocking buffer supplemented with 0.1% Tween-20, with overnight incubation at 4°C. Blots were washed three times with PBS supplemented with 0.1% Tween-20 at room temperature for 5 minutes each. The Li-Cor IRDye-labelled secondary antibodies (Mandel Scientific, Guelph, ON) were used at a dilution of 1∶10,000 in Li-Cor blocking buffer supplemented as above with 0.01% SDS and incubated at room temperature for 1 hour (ordering information and optimal dilutions for all antibodies are provided in Table S1). After washing as described, blots were scanned and analyzed with the Odyssey quantitative western blot near-infrared system (Li-Cor Biosciences, Lincoln, NE, USA) using default settings. Antibodies were initially tested individually and then grouped based on banding patterns in order to reduce the number of blots required [43]. Average band intensities were normalized by subtraction of background levels. Background normalized values are provided in Table S2 and scanned images in Figure S2. Primary and secondary antibodies were initially tested individually to identify optimal concentrations for the reduction of nonspecific banding patterns. Antibodies were then grouped where possible such that desired bands did not overlap.

### Statistical Analyses and Visualization

Data were loaded in the R statistical environment (v3.0.3) for all analyses. Protein content was aggregated across biological replicates to obtain a mean abundance with standard for each candidate protein. Aggregation into biological replicates resulted in 47 separate experimental conditions. The ratio between treated and control abundances provided the fold-difference (*M*) in expression. Individual proteins and all possible combinations of multiple proteins were assessed. Visualizations were produced using the lattice (v0.20–29) and latticeExtra (v0.6–26) R packages.

Protein content was assessed across timecourse and dose-response studies. Animals treated with TCDD were compared to control animals of the same experimental group resulting in 26–31 comparisons (some comparisons were not done due to unsatisfactory loading patterns and/or lack of sufficient sample). Differential abundance resulting from exposure to TCDD was evaluated for each candidate using an unpaired, two-tailed Student's *t*-test with Welch's adjustment for heteroscedasticity. Results were visualized as M ± standard-deviation for all experimental conditions.

Protein stability was evaluated using the NormFinder algorithm, which estimates the overall variation of a dataset by analysing its variance both within an experimental group and across experimental conditions [44]. Prior to analysis, animals were categorized into one of two groups (TCDD-treated or control) to estimate variance within experimental groups. Experiments were then split into 2 cohorts, labelled training (including experiments 1, 4, 6, 8 and 9) and validation (consisting of experiments 2, 3, 5, 7 and 10), such that each cohort contained similar types and number of animals and each cohort was analysed independently of the other. For each combination of candidates, the geometric mean of the background-normalized protein levels was calculated for each animal. For interpretation, a lower score indicates higher consistency of input across experimental groups signifying a potentially good loading control. Stability scores are available in Table S3. Linear modelling was performed to identify the contribution of each candidate protein [Y_OS_  =  α_ACTB_ + α_EEF1A1_ + α_GAPDH_ + α_HPRT_ + α_PGK1_ + α_PPIA_ + α_SDHA_ + ε] where Y_OS_ represents the overall stability of each combination of candidates and each protein is a Boolean variable indicating presence/absence in the combination while epsilon represents any error in the observations not explained by the model.

The comparative normalization method was used to contrast abundance levels between pairs of candidate molecules for each sample (adapted for use with protein abundance data from the comparative ΔC_q_ method [45]). The ability of each candidate to remove variability from other proteins was assessed and the mean standard deviation across comparisons provided a measure of stability.

mRNA analysis of candidate reference genes was reported previously for these animals and C_q_ data were downloaded (Supplementary Table 2, [31]); protein abundance and mean C_q_ data are provided in Table S4 for each animal. The correlation between protein levels and mean C_q_ values for each gene was assessed using Spearman's correlation using the AS89 method to assess statistical significance. NormFinder-generated stability scores were compared using the Spearman's correlation metric as the ordering of the scores is more meaningful than the magnitude (data available in Table S5).

**Table 2:** Summary of analysis methods.

		NormFinder		
	Student's t-test	Training	Validation	Normalization Method
**ACTB**	6/28	0.092	0.060	996.59
**EEF1A1**	11/28	0.112	0.050	278.40
**GAPDH**	5/31	0.072	0.077	316.07
**HPRT**	**1/31**	0.078	**0.046**	306.46
**PGK1**	6/29	0.144	0.081	**259.58**
**PPIA**	8/31	0.140	0.066	366.06
**SDHA**	10/26	**0.071**	0.056	286.62

Three analysis methods were used to evaluate the abundance consistency of each individual candidate protein; values in bold indicate the top ranked score for each method. 1) The difference between treated and untreated animals for each experimental condition was assessed by Student's *t*-tests; a p-value <0.05 was deemed significant. 2) The variation of each candidate was assessed using the NormFinder algorithm in two separate cohorts; a lower score indicates greater stability. 3) The comparative normalization method was used to evaluate the ability of each candidate to remove variation from a dataset; the average standard deviation for each pair-wise comparison is reported.

## Results

Quantitation of protein abundance by western blot is an essential technique widely used in the scientific community. In the past, this was typically performed using chemiluminesence. However, the Odyssey Infrared Imaging System is a well-documented alternative that provides many benefits over earlier methods, including an enhanced dynamic range of detection. Additionally, this system has the capacity for multiplexed reactions; specifically, antibodies are conjugated to IR fluorophores that can be detected at different wavelengths. As such, this system is ideal for detecting multiple targets [46].

### Univariate Analysis

A good reference gene is one whose abundance is consistent across a wide range of conditions. This is most easily detected through analysis of the fold-difference (*M*) in expression from basal levels across specific treatment conditions. Candidate abundance was compared across conditions. Moderate correlations were observed between HPRT and PGK1 (Pearson's correlation, R = 0.6) as well as EEF1A1 and SDHA (R = 0.49), while the remaining candidates were weakly correlated (Figure S3).

**Figure 1:**
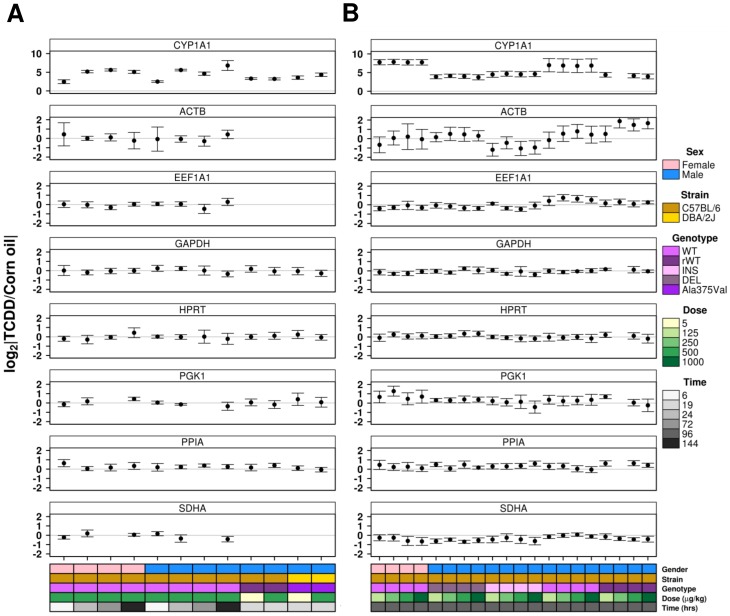
Timecourse and Dose-response by Treatment Group. The fold-differences in protein abundance between treated and control animals were calculated and results compared across all conditions. (**A**) Timecourse and (**B**) dose-response studies were visualized. Points represent the fold change in abundance (in log_2_ space) and error bars indicate the standard deviation for each experimentally unique group.

To better understand this variation, each experimental group was examined individually (Figure 1). Of the 31 different experiment groups and 192 animals for which protein data were obtained (and for which mRNA data were obtained previously), HPRT was significantly altered by TCDD in only one group and GAPDH (5/31 conditions significantly altered) was also consistent, while the remainder of targets displayed less consistency, with greater than 20% of conditions altered (Table 2). To verify our samples and approach, the prototypical *Ahr*-regulated gene, CYP1A1, was examined as above and was determined to be significantly altered by TCDD at the protein level across all 31 conditions, as expected (Figure 1).

**Figure 2:**
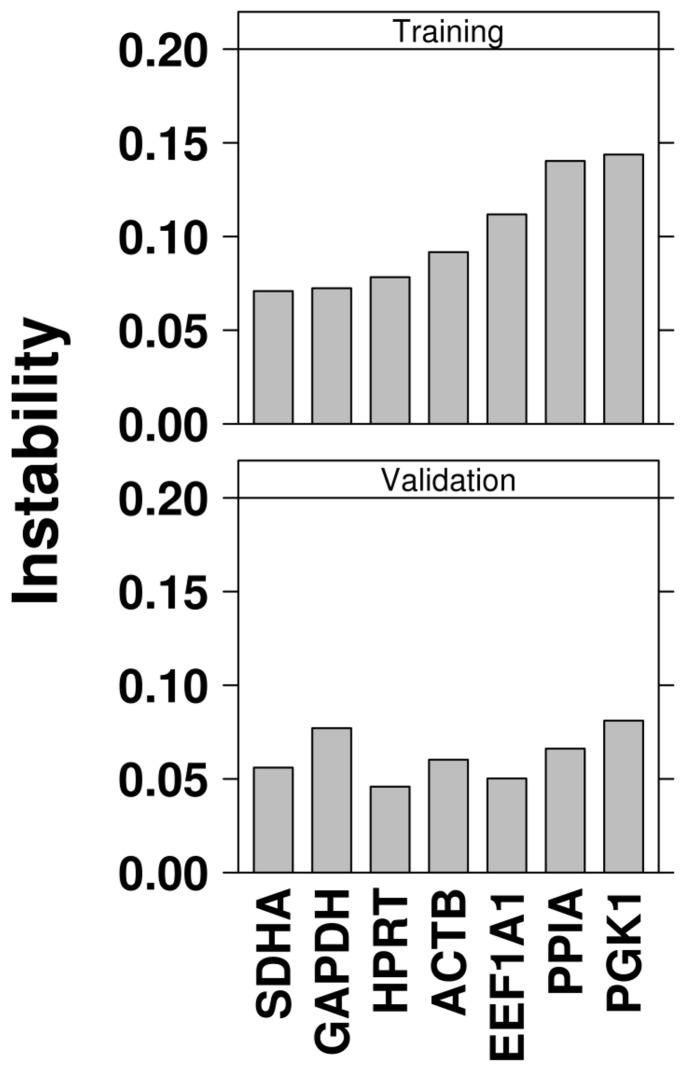
Univariate Analysis of Candidate Stability. Animals were separated into training and validation cohorts based on experiment, ensuring similar treatment conditions and animal numbers appeared in both sets. Within each cohort, animals were categorized as either TCDD-treated or control. Candidate proteins were analyzed using the NormFinder algorithm to determine stability across all treatment groups. A lower value indicates less variance across all experimental conditions.

As this evaluation of differences in TCDD-altered abundance only accounts for variation within a single treatment, individual candidate stability across all experimental conditions was assessed using the NormFinder algorithm [44]. Briefly, NormFinder estimates the overall numerical stability of a molecule based on variability within a single treatment condition, variation within and between multiple conditions and systemic variation between experimental runs. Lower stability scores indicate less variation while higher scores indicate greater instability across experiments. As with our previous analysis of reference genes for transcriptomic analysis [31], experiments were organized into training and validation sets, thereby evaluating protein stability in two independent cohorts (Figure 2, Table 2). Although the cohorts differed in the magnitudes of stability scores, HPRT and SDHA were consistently amongst the most stable of the candidates, while PGK1 and PPIA were consistently the most unstable of the proteins evaluated.

To ensure that our results are not confounded by a shift in abundance caused by technical variation and independent of TCDD-treatment, we applied an alternate univariate analysis technique. Under typical experimental settings, it would be the purpose of the reference gene to normalize abundance levels for this shift. To this end, abundances of 6 proteins from each animal were normalized using the 7^th^, and the variance across technical replicates evaluated. This process was repeated using each protein as the normalization candidate. Using this approach, a lower score indicated greater stability across a dataset resulting from normalization with the given candidate protein (Table 2). By this method, PGK1 and EEF1A1 were determined to be the most stable of candidate proteins while ACTB was responsible for increased variation, likely due to the difference in magnitude of the intensity values between targets (intensity values for ACTB are significantly higher than for other candidates). Surprisingly, while PGK1 was identified as one of the more variable candidates both by analysis of fold-differences and the NormFinder algorithm, it was among the most stable candidates by this normalization method.

### Multivariate Analysis

**Figure 3:**
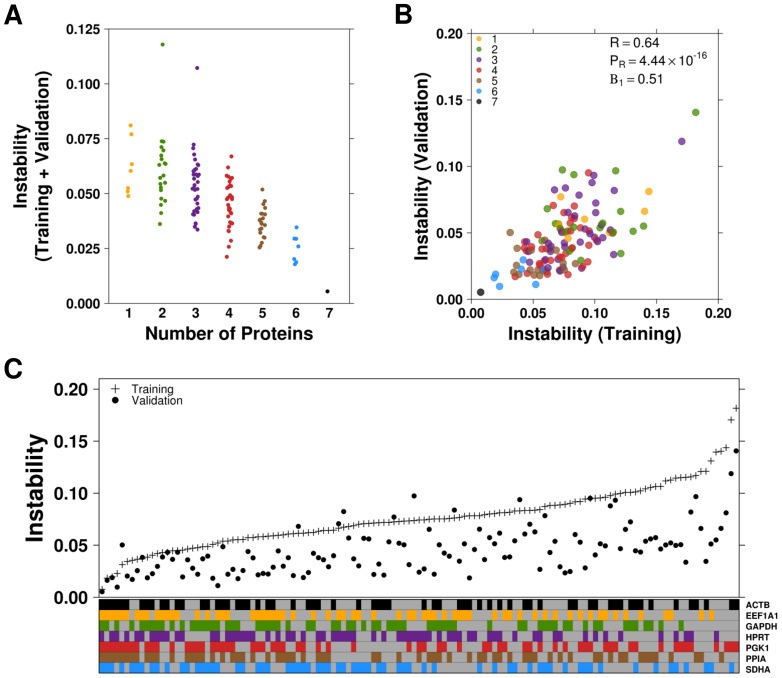
Multivariate Analysis of Candidate Stability. Animals were categorized as either TCDD-treated or control and separated into training and validation cohorts. All possible combinations of candidates were analyzed using the NormFinder algorithm. A lower value indicates less variance across all experimental conditions. (**A**) Combinations of candidates were organized according to the number of proteins included, in order to determine the optimal number of proteins used. (**B**) Stability results for each combination of candidates were compared between the training and validation sets to assess concordance. (**C**) Results for each combination of gene(s) were plotted for both the training (+) and validation (•) cohorts. Combinations are organized according to performance in the training set.

It has previously been shown that the use of multiple reference genes can improve normalization [31], [47]. Although this generally applies to more high-throughput technologies capable of analyzing a large number of genes simultaneously, we evaluated the usefulness of utilizing multiple controls for western blot studies. The normalization capabilities of each possible combination of our candidate proteins were tested using the NormFinder algorithm, as described above. In general, including more control genes improved stability; however, specific pairs of candidates, and even some individual candidates, showed greater stability than some larger combinations (Figure 3A). Within each subset of samples, candidate combinations generally performed similarly; however, the training cohort demonstrated slightly more variance among samples (Pearson's correlation  = 0.64) (Figure 3B). Despite this, the combination of all 7 candidates displayed the greatest stability in both cohorts (Figure 3C).

**Figure 4:**
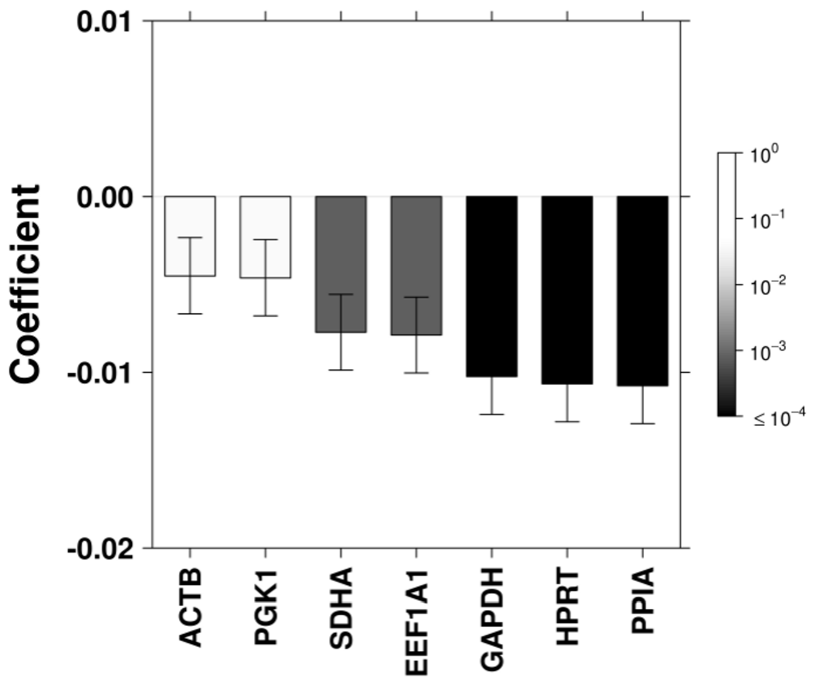
Linear Modelling of Multivariate Results. Linear modelling was performed to identify the contribution of each candidate to stability as determined by NormFinder across the complete dataset bars are coloured according to FDR-corrected p-value; error bars indicate standard error within the model; negative values are representative of decreased variation (increased stability).

As a greater instability score appeared to primarily result from the inclusion of select candidates, linear modeling was performed to examine the contribution of each candidate to overall stability. ACTB and PGK1 decreased stability while GAPDH, HPRT and PPIA significantly increased stability (Figure 4).

### Comparison with mRNA

**Figure 5:**
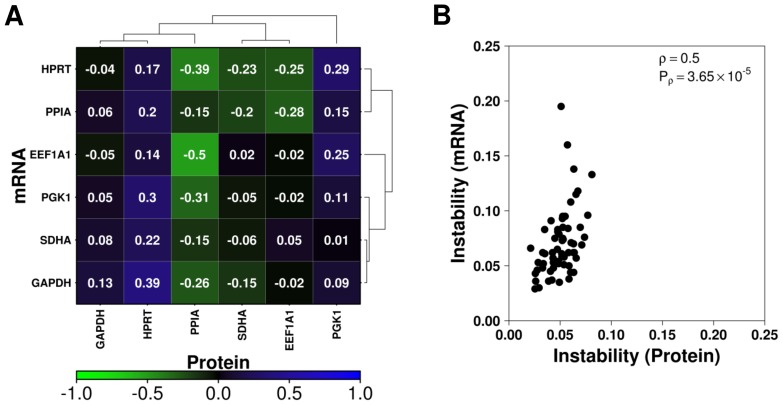
Comparison of candidate mRNA and protein abundances. mRNA and protein abundances as determined by qPCR and quantitative western blot were compared for each candidate. (**A**) Spearman's correlation was used to compare mean C_q_ values across technical replicates for qPCR and protein intensity for candidate genes and visualized in a heatmap organized using divisive clustering: blue indicates perfect correlation, green indicates inverse correlation and black indicates little or no correlation. Note that an increasing mRNA abundance results in a lower C_q_; hence an inverse correlation indicates similarity between molecule abundances. (**B**) Spearman's correlation was used to assess similarity in candidate combination stability calculated by NormFinder for each data type.

As a similar analysis on the mRNA abundance of these candidates had been previously conducted in the liver of these animals, we thus compared the mRNA and protein abundances for each candidate. Spearman's correlation was used to determine whether protein abundance was concordant with mRNA levels. In general, there was little to no correlation between these molecules, possibly indicating differential regulation of translational mechanisms or variation in stability of the protein (Table 3, Figure 5A). To verify this, stability scores for each dataset generated by NormFinder were combined, and the overlapping gene combinations compared (Figure 5B). Interestingly, while the abundance patterns of these candidates varied, combinations of candidates generally demonstrated similar stability (Spearman's correlation  = 0.5, p = 3.65×10^−5^). Among the candidates (independently or in combination) that overlapped between studies, HPRT was among the most stable individual genes while the partnership of HPRT and GAPDH was consistently the most stable pair of candidates. Beyond this, the order of combination stability varied, sometimes dramatically, between data types. For example, the combination of EEF1A1, GAPDH and PPIA proved highly stable within the mRNA data, but was among the most unstable within the protein dataset. Alternatively, the pair-wise combination of EEF1A1 and PGK1 was among the most stable within the protein data and among the least stable in the mRNA data (Table S5).

**Table 3:** Comparison between mRNA and protein abundances.

	Spearman's correlation	
	ρ	p-value
**EEF1A1**	−0.02	0.79
**GAPDH**	0.13	0.08
**HPRT**	0.17	0.02
**PGK1**	0.11	0.14
**PPIA**	−0.15	0.04
**SDHA**	−0.06	0.46

Spearman's correlation was used to evaluate concordance between mRNA and protein abundances as determined by qPCR (mean C_q_ of technical replicates) and western blot (log_2_ of the protein intensity). Note that an increasing mRNA abundance results in a lower C_q_; hence an inverse correlation indicates similarity between molecule abundance.

## Discussion

Thorough validation of reference genes is essential prior to any quantitative experimentation. Whether for evaluation of mRNA or protein abundance, all experimental methods are prone to some variation; the general rule is that each step in a process will introduce some error. This error may not be noticeable throughout the process, and only becomes apparent in downstream analyses, such as molecule quantitation. To ensure accurate interpretation, it is imperative to account for this technical variation. Estimation of target values relative to a reference molecule, whether internal or exogenous spike-in control is a proven method across technologies [48], [49]. In the case of an endogenous molecule reference, careful validation must first occur as it has been shown that even classically-used controls can differ in abundance across different sample types or even by sample handling methods. For example, *Gapdh* was found to be less stable over time in FFPE breast tumour samples by qRT-PCR [50] whereas it was deemed a suitable reference gene for use in lung tumour FFPE samples [51]. In a proteomic analysis, multiple species of GAPDH were identified within human platelet samples; of these, the most abundant of species was highly variable across both age and sex [52]. This indicates that particular effort must be made when validating loading controls for western blot, as different antibodies may target different species.

Exposure to TCDD has been shown to have a dramatically different effect on transcriptomic regulation across various animal models. This has been shown to result from ligand activation of the AHR by TCDD-binding [8] while the degree of toxicity is directly related to the *Ahr*-genotype within rodents. While studies into the specific transcriptomic changes responsible for overall toxicity are still ongoing, progress has been made in the identification of candidate lists within various animal models, including strains of rats [53], [54] and mice [55]. However, as toxicity likely results from subsequent changes in the proteome, further studies are required to verify which of these candidate genes are concomitantly altered at the protein level. While validation of reference genes for RNA quantitation in various mouse models has been completed [31], there is no reason to expect similar results to be obtained at the level of the proteome.

Here, we have evaluated the protein abundance of 7 reference genes for use in toxico-proteomic analyses of TCDD-induced toxicity within a wide range of mouse models. In particular, we have assessed the effect of TCDD exposure on protein abundance within mouse models of various strains, *Ahr*-genotype and sex across both a timecourse and dose-response approach. Protein abundance was assessed by quantitative western blot analysis and each candidate's suitability as a reference control was evaluated using 3 analysis methods: 1) the fold-difference in protein content from basal levels, 2) the NormFinder algorithm [44], which is an assessment of target stability and 3) the ability of each candidate to reduce instability of the others [45].

As TCDD is known to have a significant impact on transcriptional regulation, and has been shown to affect the proteome [56], the protein abundance of our candidates was first assessed using biologically similar animals that were treated with either TCDD (at various doses) or corn oil alone. HPRT was identified as the protein least affected by TCDD while EEF1A1 and SDHA showed significant variability across multiple experimental conditions (Figure 1, Table 2). The suitability of this method is proven through the evaluation of CYP1A1; a protein involved in the detoxification of xenobiotics known to be significantly induced by TCDD. As well, since data for both treated and control animals were generated on a single western blot (experiencing identical experimental settings), this metric was arguably the most appropriate for our goals. Next, as the purpose of a reference gene is to efficiently remove technical variation from the quantified results, we sought to characterize the residual variability among the remaining proteins after normalization with each candidate. An assumption of this method is that all candidate proteins demonstrate consistent expression over experimental conditions and that increased variation indicates decreased stability of the candidate in question [47]. Here we identified EEF1A1 and PGK1 as the most consistently expressed candidate genes while PPIA was again determined to be the least stable candidate (Table 2). The high instability of ACTB should be interpreted with caution as it does not follow the above assumption. One limitation of this approach is its disregard for technical considerations; since each western blot contained a separate experiment, and were performed one at a time, some technical variation would be inherent across the entire study. Finally, unlike the above comparative method, the NormFinder algorithm considers variation both within and between experiments in its assessment of candidate stability [44]. While the specific order of stability varied, NormFinder analysis identified HPRT, ACTB and SDHA as the most stable candidates in all cohorts examined (training, validation and overall). Similarly, PGK1 and PPIA were always deemed the most unstable candidates. The consistency in stability scores for each candidate protein verifies that NormFinder is a robust and reproducible method for identifying good reference proteins.

A major finding of our previous study of reference gene stability in qPCR studies was that greater stability was obtained through increasing the number of reference genes used. This finding was consistent with other reference gene validation studies [47], [57]. In order to determine whether this finding was consistent with proteomic analysis, NormFinder analysis was applied as above. In general, the trend of increasing stability was consistent with the inclusion of an increasing number of candidates (Figure 3). However, due to the low-throughput nature of any western blot analysis, increasing the number of reference proteins is largely impractical. Therefore, careful selection of 2 or 3 candidates with good stability would prove ideal. In some cases, even a single reference gene could provide a more stable normalization factor than a larger, less consistently expressed group of candidates. To this effect, linear modelling of the multivariate analysis indicated that 2 of the 3 most stable candidates identified in the univariate analysis (HPRT and SDHA) each contributed significantly to increased stability when included in combinations of any number of candidates (Figure 4) while PGK1 contributed less.

The availability of both mRNA and protein abundances collected from the same 192 animals presented an interesting opportunity, as an in-depth comparison of these molecules for these candidate genes across such a wide range of conditions has yet to be performed. We sought to determine whether targets selected as optimal reference genes at the level of mRNA would be suitable for normalization of protein abundance data. A comparison of abundance levels suggested little or no correlation between molecules (Table 3). The largest correlation coefficient, though showing an inverse relationship in abundance, was observed for HPRT. While analysis of the fold-changes identified HPRT as most stable univariate candidate at the protein level, it was much less stable at the level of mRNA abundance. However, it consistently ranked among the most stable genes across all analysis methods in each study. Alternatively, the least stable gene identified in the RNA study, *Sdha*, ranked among the most stable in the current protein analysis and did not show correlation between molecules. As such, the optimal reference gene for studies of mRNA abundance may not be optimal for studies of protein abundance and should be validated prior to use. Conversely, multivariate analysis by NormFinder generated stability scores that were moderately correlated between data types and, in general, these scores improved with the addition of an increased number of genes. Even so, the practicality of using a larger number of genes is limited by the technology used and must be taken into consideration. As such, while using a larger number of genes is encouraged for studies easily multiplexed (such as qPCR), careful selection of fewer genes is required for low-throughput methods such as western blot.

For any type of quantitative analysis, data must be thoroughly normalized in order to account for the technical variation inherent in any experiment and to ensure reliable and reproducible results. The use of multiple controls is ideal for generation of a normalization factor; however, a carefully selected group of fewer candidates can prove sufficient when larger numbers are impractical. Here we have identified and suggested specific combinations of loading controls, such as HPRT alone or combined with ACTB or GAPDH, for use in western blot analysis of various mouse models of TCDD toxicity.

## Supporting Information

Figure S1 **Experimental Design.** Mice were treated with either 0, 5, 125, 250, 500 or 1000 µg/kg TCDD dissolved in corn oil vehicle and euthanized at 6, 19, 24, 72, 96 or 144 hours post-exposure. Timecourse experiments followed male (blue) and female (pink) wild-type C57BL/6 mice treated with 500 µg/kg TCDD. Male DBA/2J and ratonized-WT mice were collected at 19 hours post-exposure following treatment with either 5 or 500 µg/kg TCDD. Dose-response experiments followed male (blue) wild-type or ratonized mice and female (pink) wild-type mice treated with a single dose of TCDD and euthanized 96 hours following exposure.
(PPT)

Figure S2 **Western blots.** Western blots were scanned and analyzed with the Odyssey quantitative western blot near-infrared system using default settings. Each blot was scanned twice as two groups of antibodies were used. Wells with unusual loading patterns (noted by the *) were not used in the downstream analysis.
(PPT)

Figure S3 **Correlation of Candidate Proteins.** The fold-difference in abundance between treated and control groups were calculated for each experimental condition and Pearson's correlations applied. Correlation results were visualized using a heatmap and organized by divisive clustering. Blue indicates perfect correlation; green represents inverse correlations while black indicates little or no correlation. Pearson's correlations are shown in white for each pair-wise comparison.
(TIF)

Figure S4 **Ponceau Stain.** Total protein abundance was assessed in a representative gel using Colloidal Blue Stain pre- (A) and post-transfer (B). Total protein was quantified and background-normalized intensity values were visualized for both gels (C). Transferred protein was also visualized on the membrane (D) using Ponceau stain. Lanes labelled in black indicate untreated samples, while blue labels are TCDD-treated (500 µg/kg) samples. The first four lanes show increasing amounts of loaded protein.
(PPTX)

Table S1 **Antibody Information.**
(XLS)

Table S2 **Protein Abundances.**
(XLSX)

Table S3 **NormFinder Stability Scores.**
(XLS)

Table S4 **Comparison of mRNA and Protein Abundances.**
(XLS)

Table S5 **Comparison of mRNA and Protein Stability Scores.**
(XLS)

Table S6 **Animal Information.**
(XLS)
