# Supplementary figures and images for: Identification of Reference Proteins for Western Blot Analyses in Mouse Model Systems of 2,3,7,8-Tetrachlorodibenzo-P-Dioxin (TCDD) Toxicity
Source: PLoS One. 2014 Oct 17;9(10):e110730. doi: 10.1371/journal.pone.0110730 (PMC4201576; doi:10.1371/journal.pone.0110730)

## Slide 1
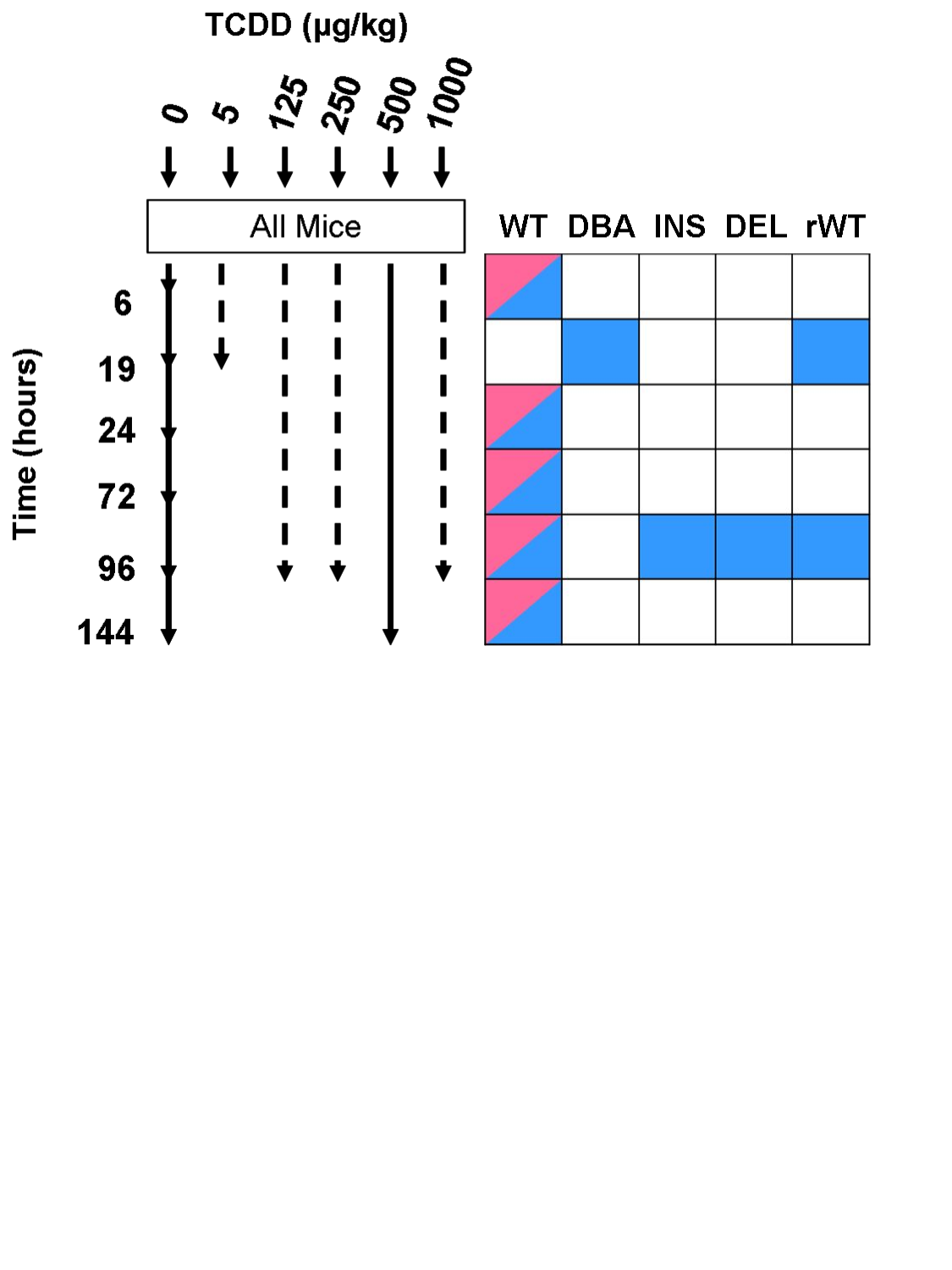

Supplement: Figure S1 — Experimental Design. Mice were treated with either 0, 5, 125, 250, 500 or 1000 µg/kg TCDD dissolved in corn oil vehicle and euthanized at 6, 19, 24, 72, 96 or 144 hours post-exposure. Timecourse experiments followed male (blue) and female (pink) wild-type C57BL/6 mice treated with 500 µg/kg TCDD. Male DBA/2J and ratonized-WT mice were collected at 19 hours post-exposure following treatment with either 5 or 500 µg/kg TCDD. Dose-response experiments followed male (blue) wild-type or ratonized mice and female (pink) wild-type mice treated with a single dose of TCDD and euthanized 96 hours following exposure. (PPT) [file pone.0110730.s001.ppt]

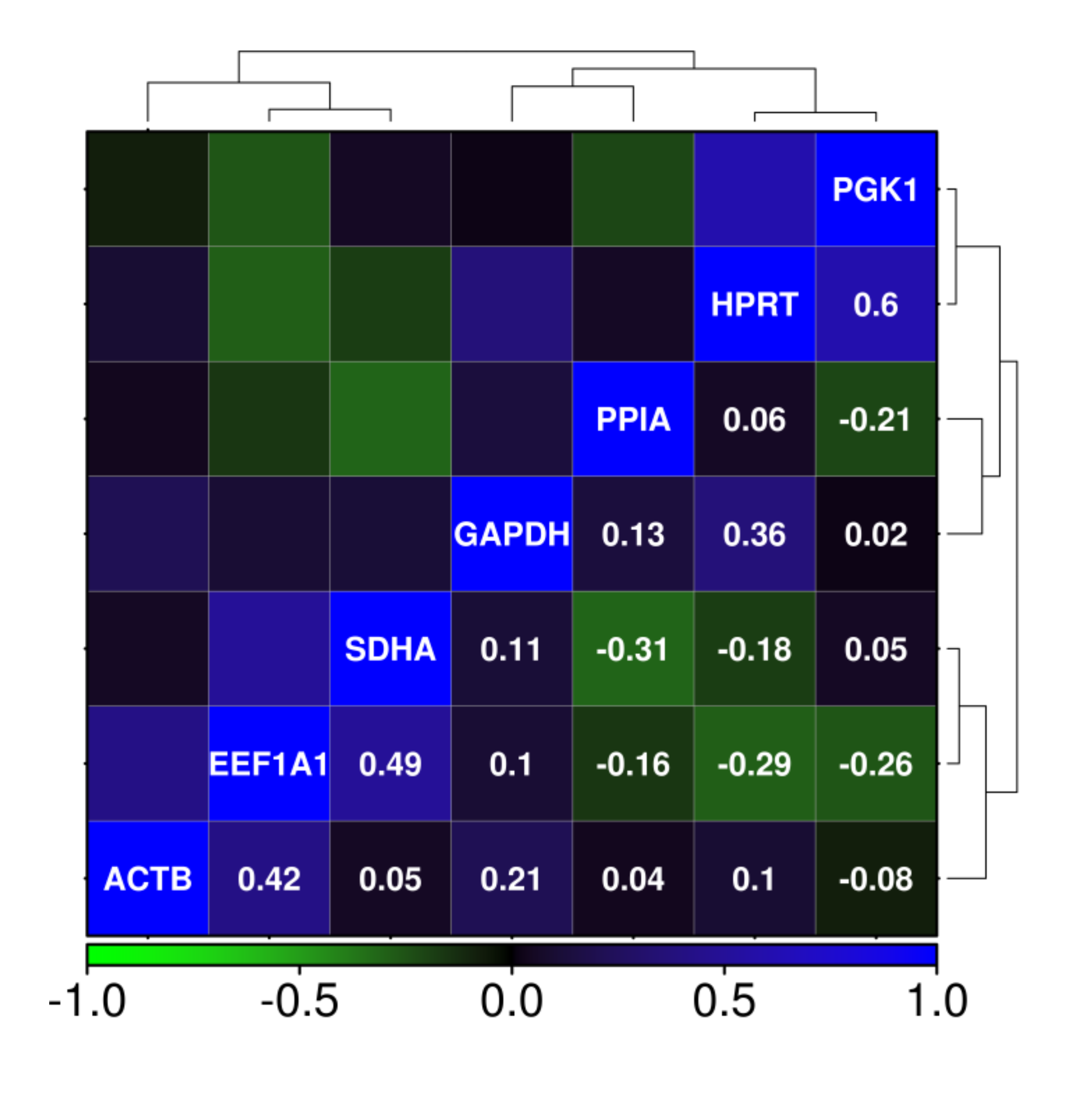

Supplement: Figure S3 — Correlation of Candidate Proteins. The fold-difference in abundance between treated and control groups were calculated for each experimental condition and Pearson's correlations applied. Correlation results were visualized using a heatmap and organized by divisive clustering. Blue indicates perfect correlation; green represents inverse correlations while black indicates little or no correlation. Pearson's correlations are shown in white for each pair-wise comparison. (TIF) [file pone.0110730.s003.tif]

## Slide 1
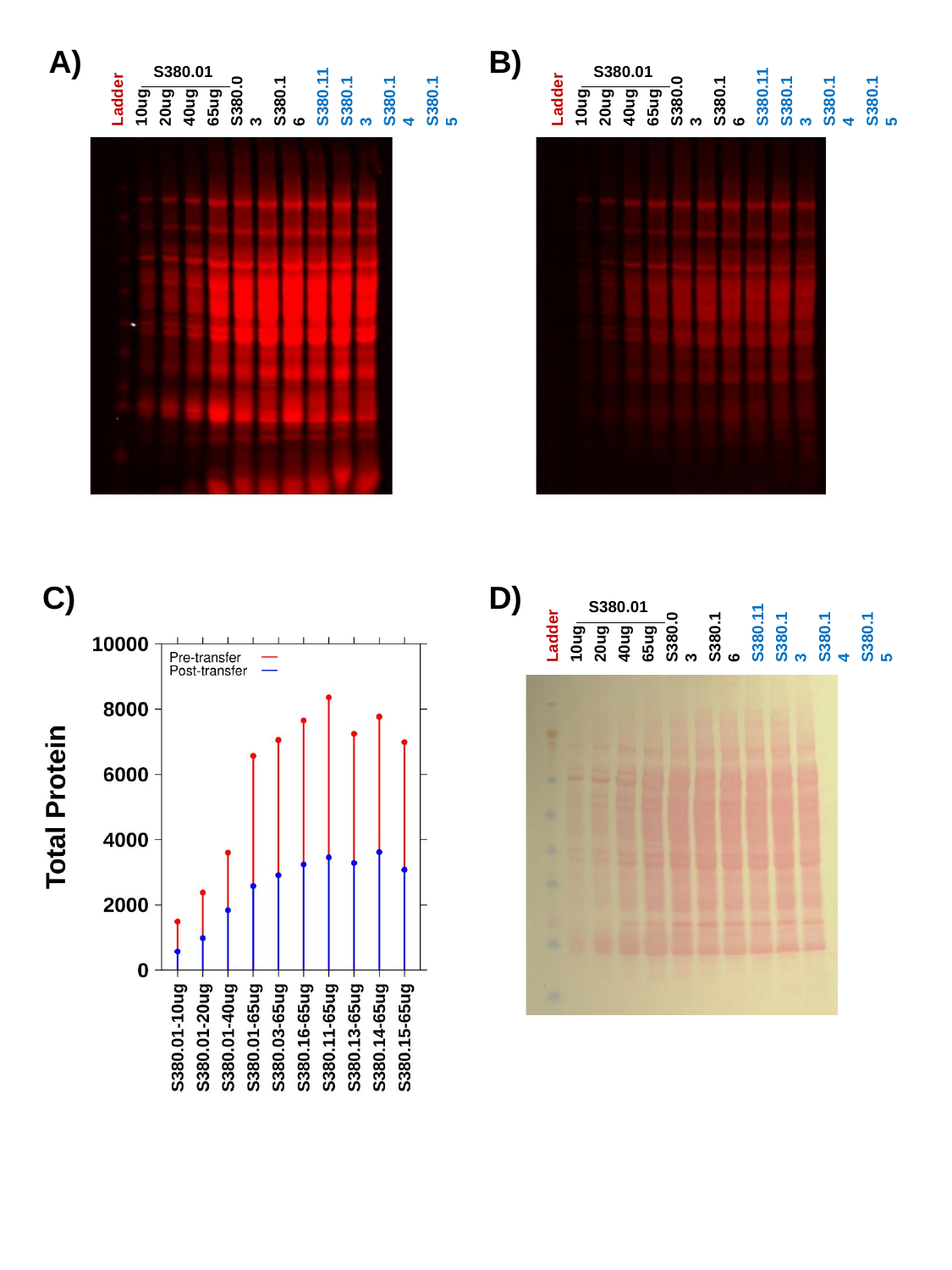

A)
B)
Ladder
10ug
20ug
40ug
65ug
S380.03
S380.16
S380.11
S380.13
S380.14
S380.15
| S380.01 |
| --- |
Ladder
10ug
20ug
40ug
65ug
S380.03
S380.16
S380.11
S380.13
S380.14
S380.15
| S380.01 |
| --- |
C)
D)
Ladder
10ug
20ug
40ug
65ug
S380.03
S380.16
S380.11
S380.13
S380.14
S380.15
| S380.01 |
| --- |

Supplement: Figure S4 — Ponceau Stain. Total protein abundance was assessed in a representative gel using Colloidal Blue Stain pre- (A) and post-transfer (B). Total protein was quantified and background-normalized intensity values were visualized for both gels (C). Transferred protein was also visualized on the membrane (D) using Ponceau stain. Lanes labelled in black indicate untreated samples, while blue labels are TCDD-treated (500 µg/kg) samples. The first four lanes show increasing amounts of loaded protein. (PPTX) [file pone.0110730.s004.pptx]
