# Supplementary material for: Identification of Reference Proteins for Western Blot Analyses in Mouse Model Systems of 2,3,7,8-Tetrachlorodibenzo-P-Dioxin (TCDD) Toxicity
Source: PLoS One. 2014 Oct 17;9(10):e110730. doi: 10.1371/journal.pone.0110730 (PMC4201576; doi:10.1371/journal.pone.0110730)
Supplement: Figure S2 — Western blots. Western blots were scanned and analyzed with the Odyssey quantitative western blot near-infrared system using default settings. Each blot was scanned twice as two groups of antibodies were used. Wells with unusual loading patterns (noted by the *) were not used in the downstream analysis. (PPT) [file pone.0110730.s002.ppt]

## Slide 1
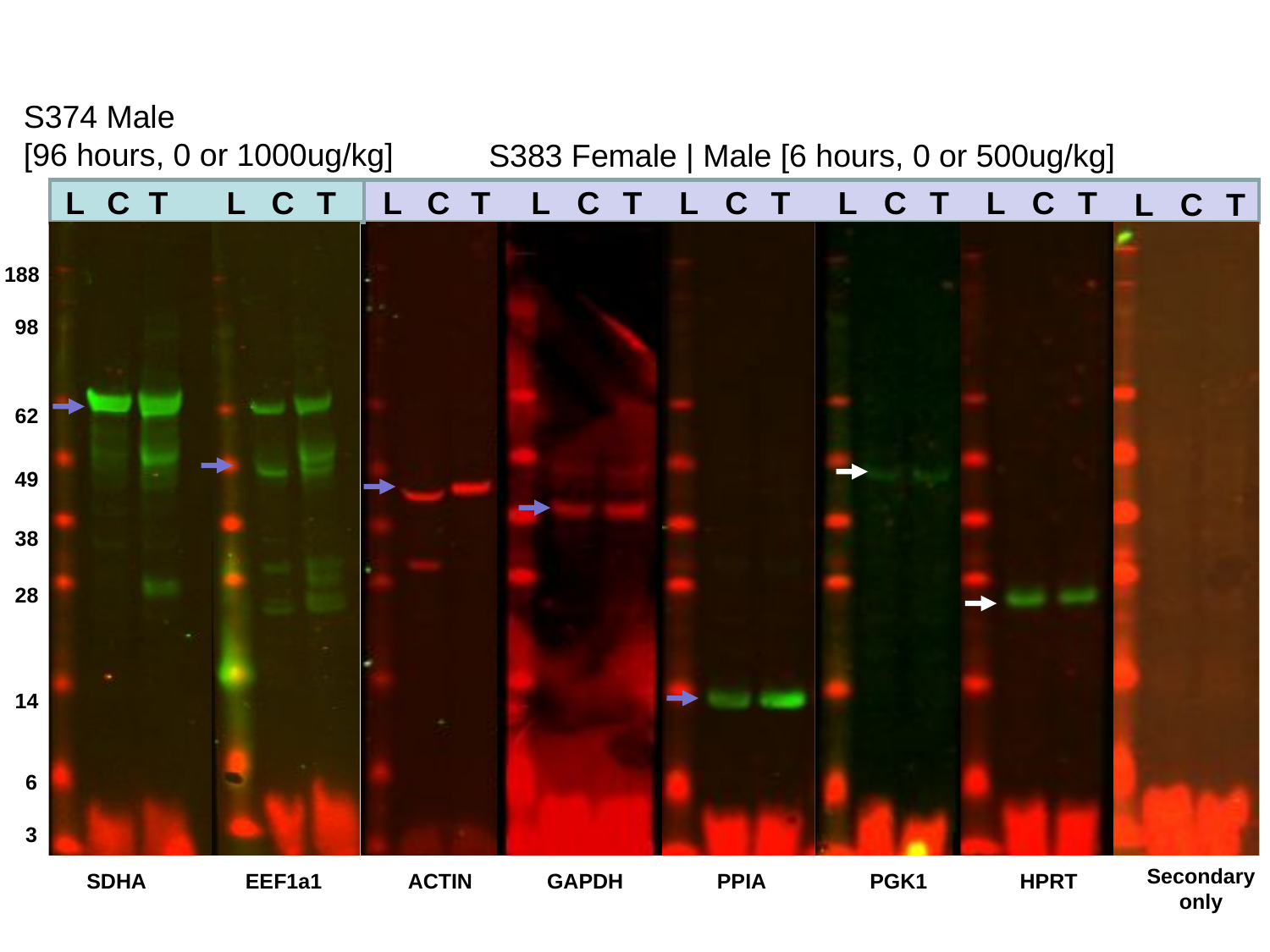

S374 Male
[96 hours, 0 or 1000ug/kg]
S383 Female | Male [6 hours, 0 or 500ug/kg]
| L | C | T |
| --- | --- | --- |
| L | C | T |
| --- | --- | --- |
| L | C | T |
| --- | --- | --- |
| L | C | T |
| --- | --- | --- |
| L | C | T |
| --- | --- | --- |
| L | C | T |
| --- | --- | --- |
| L | C | T |
| --- | --- | --- |
| L | C | T |
| --- | --- | --- |
188
98
62
49
38
28
14
6
3
Secondary only
GAPDH
PPIA
PGK1
HPRT
SDHA
EEF1a1
ACTIN

## Slide 2
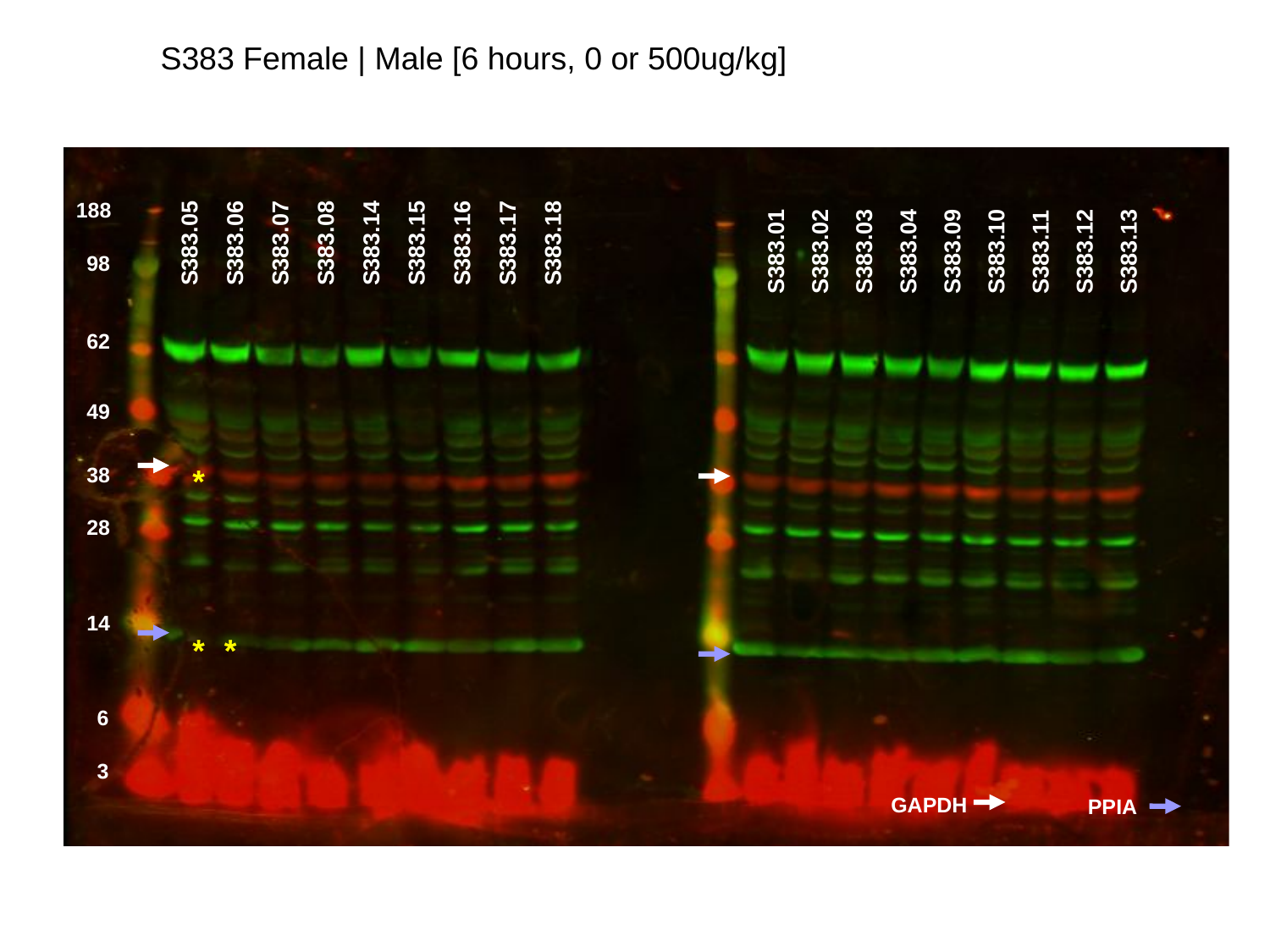

S383.05
S383.06
S383.07
S383.08
S383.14
S383.15
S383.16
S383.17
S383.18
S383.01
S383.02
S383.03
S383.04
S383.09
S383.10
S383.11
S383.12
S383.13
S383 Female | Male [6 hours, 0 or 500ug/kg]
188
98
62
49
38
*
28
14
*
*
6
3
GAPDH
PPIA

## Slide 3
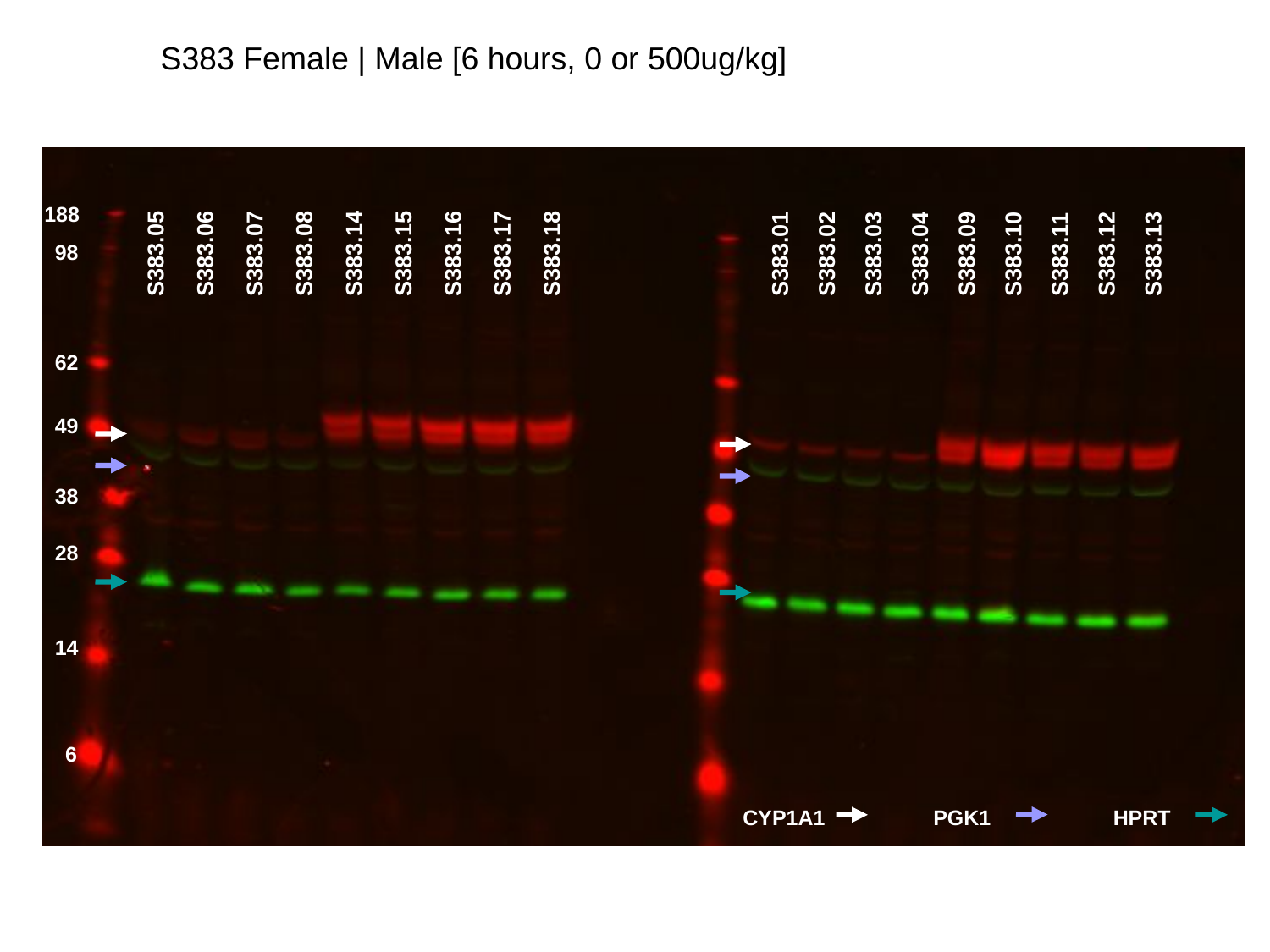

S383.05
S383.06
S383.07
S383.08
S383.14
S383.15
S383.16
S383.17
S383.18
S383.01
S383.02
S383.03
S383.04
S383.09
S383.10
S383.11
S383.12
S383.13
S383 Female | Male [6 hours, 0 or 500ug/kg]
188
98
62
49
38
28
14
6
CYP1A1
PGK1
HPRT

## Slide 4
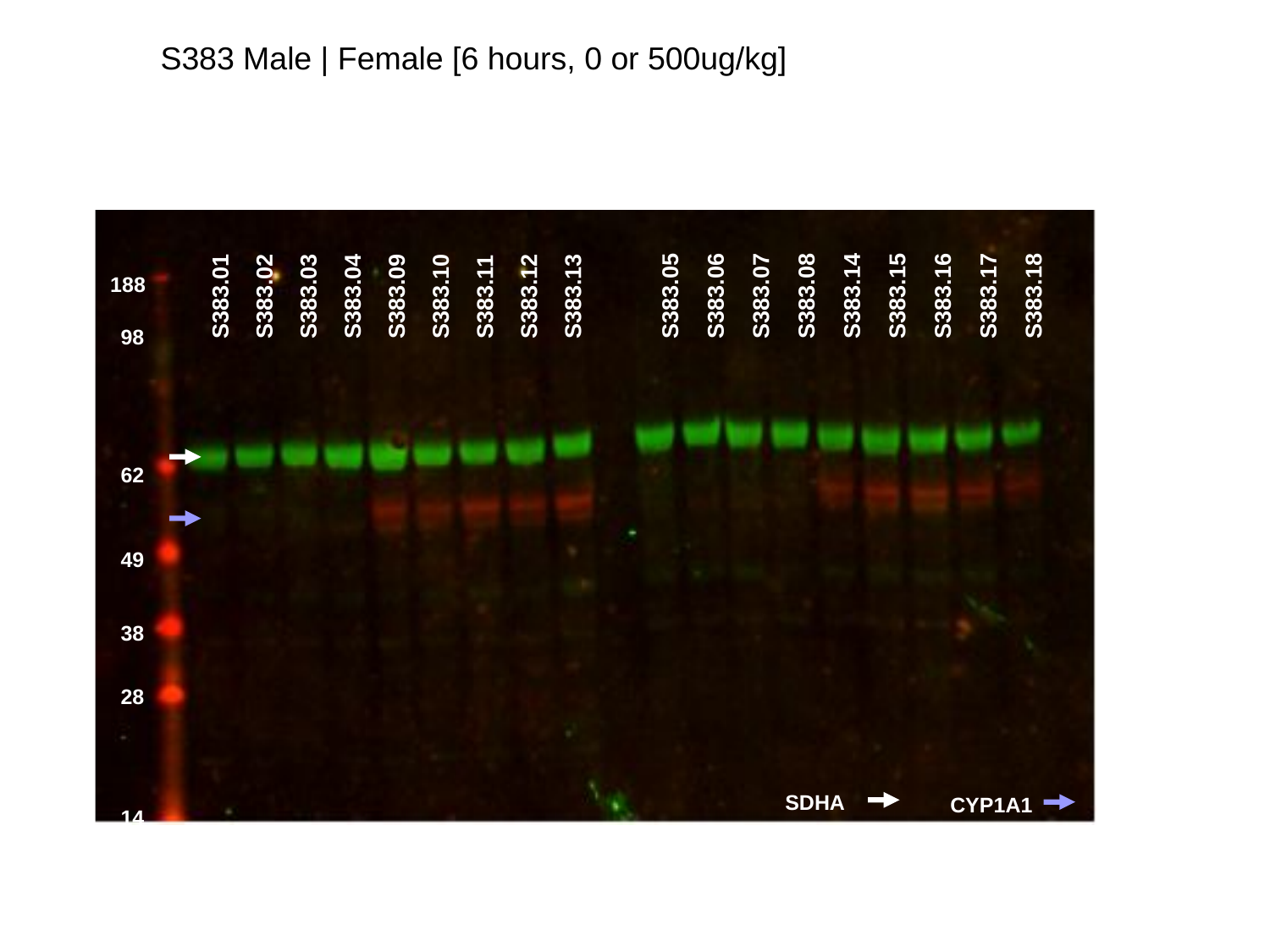

S383 Male | Female [6 hours, 0 or 500ug/kg]
S383.05
S383.06
S383.07
S383.08
S383.14
S383.15
S383.16
S383.17
S383.18
S383.01
S383.02
S383.03
S383.04
S383.09
S383.10
S383.11
S383.12
S383.13
188
98
62
49
38
28
SDHA
CYP1A1
14

## Slide 5
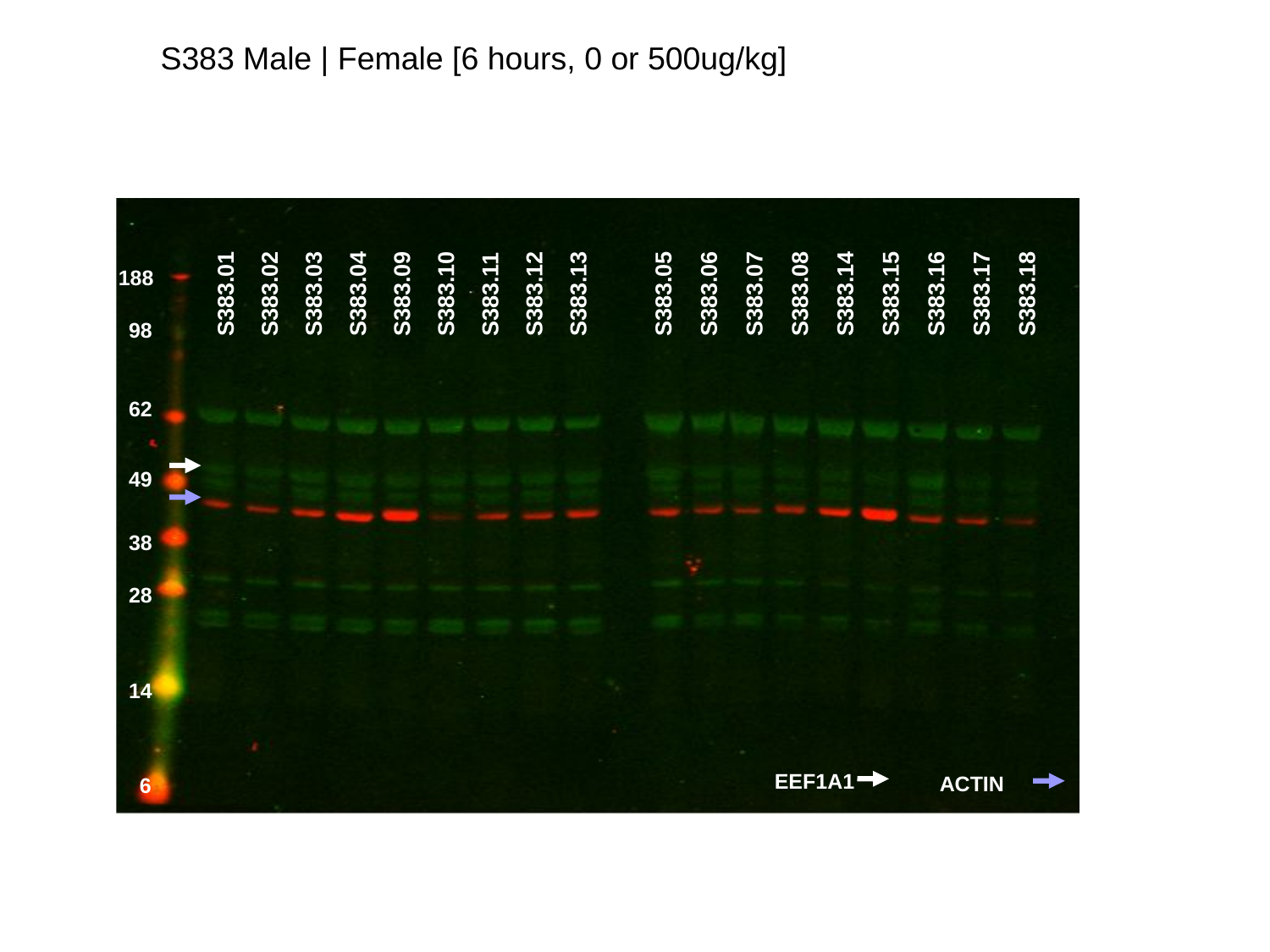

S383 Male | Female [6 hours, 0 or 500ug/kg]
S383.05
S383.06
S383.07
S383.08
S383.14
S383.15
S383.16
S383.17
S383.18
S383.01
S383.02
S383.03
S383.04
S383.09
S383.10
S383.11
S383.12
S383.13
188
98
62
49
38
28
14
EEF1A1
ACTIN
6
3

## Slide 6
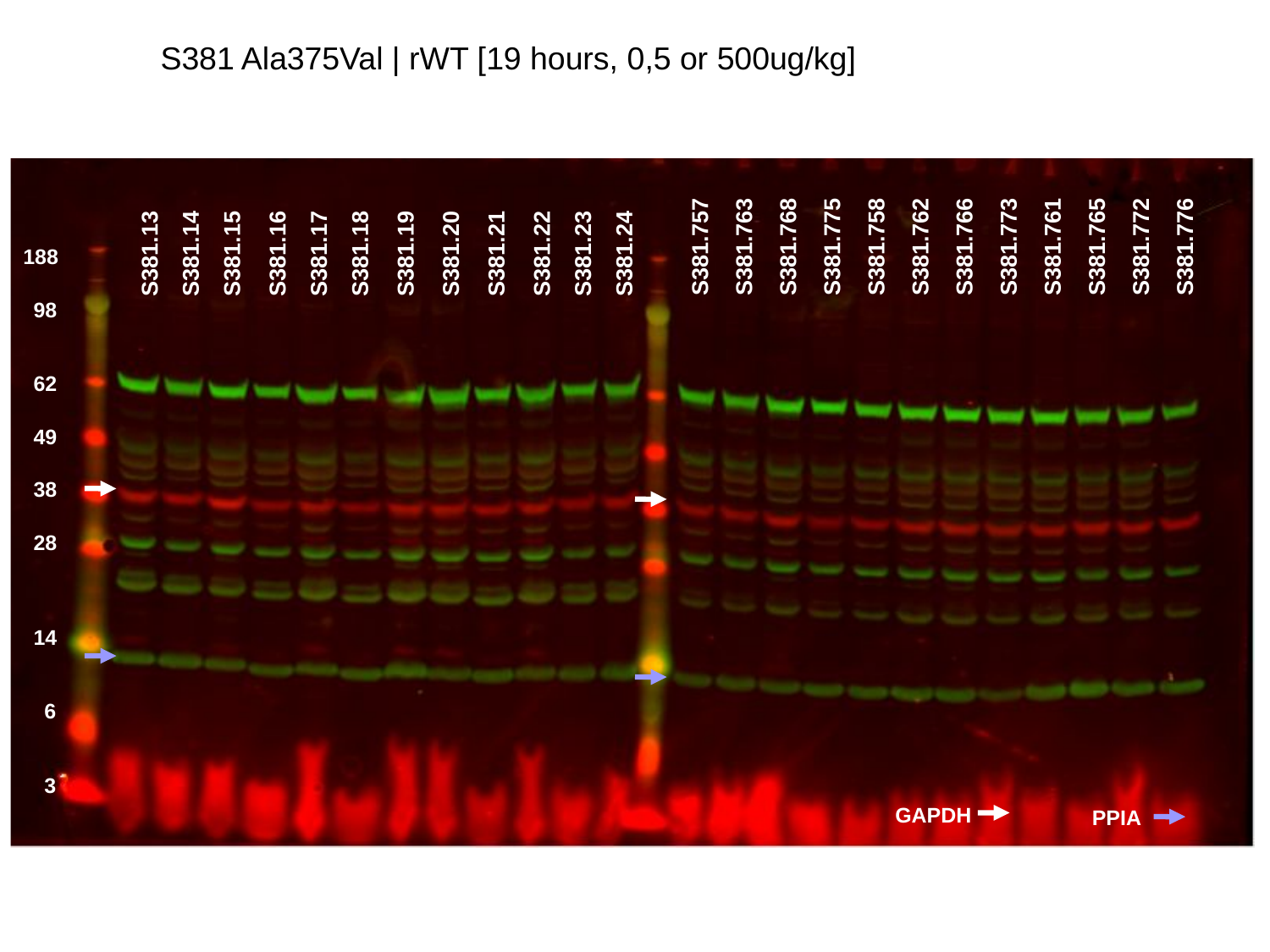

S381.757
S381.763
S381.768
S381.775
S381.758
S381.762
S381.766
S381.773
S381.761
S381.765
S381.772
S381.776
S381.13
S381.14
S381.15
S381.16
S381.17
S381.18
S381.19
S381.20
S381.21
S381.22
S381.23
S381.24
S381 Ala375Val | rWT [19 hours, 0,5 or 500ug/kg]
188
98
62
49
38
28
14
6
3
GAPDH
PPIA

## Slide 7
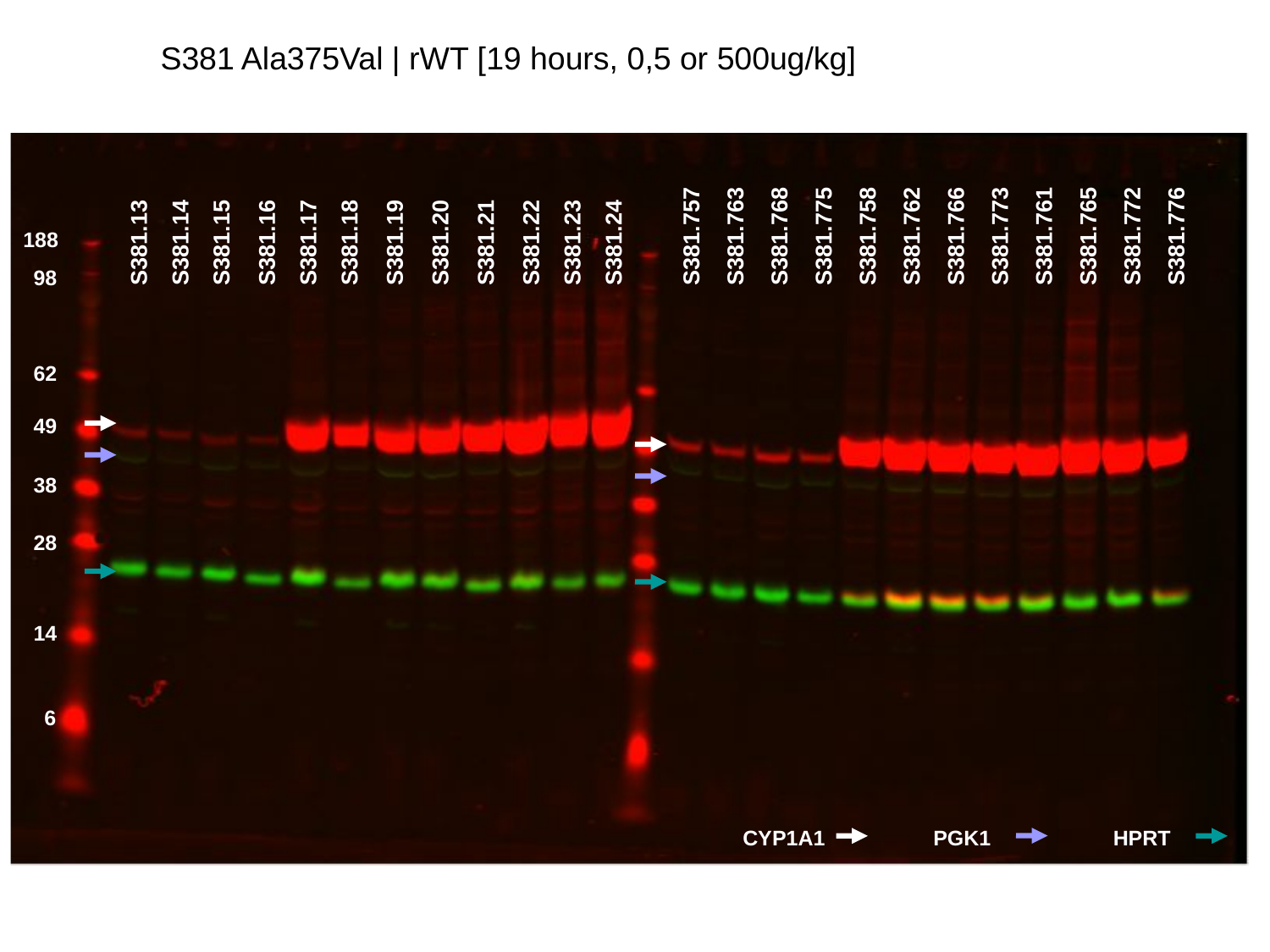

S381.757
S381.763
S381.768
S381.775
S381.758
S381.762
S381.766
S381.773
S381.761
S381.765
S381.772
S381.776
S381.13
S381.14
S381.15
S381.16
S381.17
S381.18
S381.19
S381.20
S381.21
S381.22
S381.23
S381.24
S381 Ala375Val | rWT [19 hours, 0,5 or 500ug/kg]
188
98
62
49
38
28
14
6
CYP1A1
PGK1
HPRT

## Slide 8
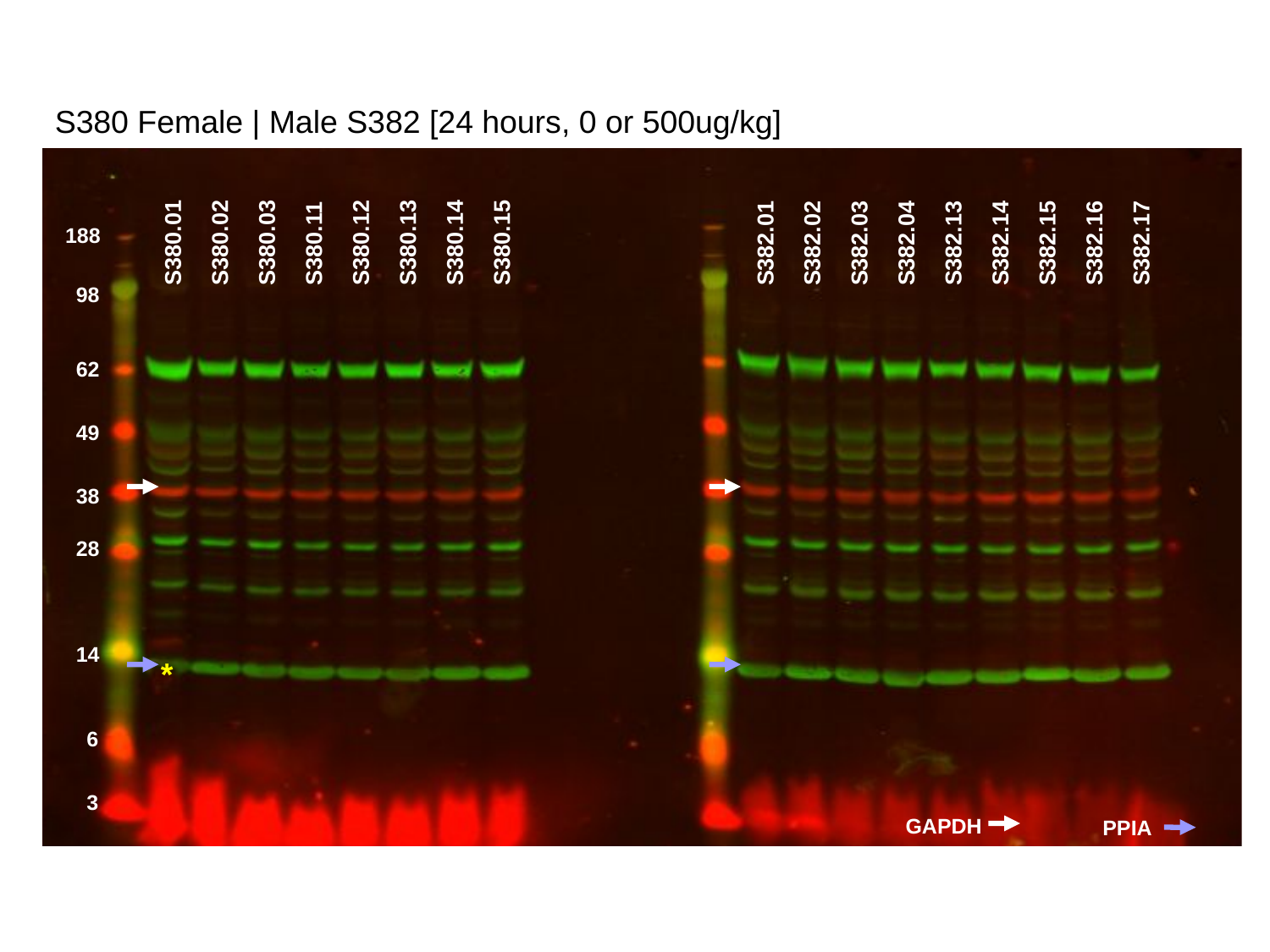

S382.01
S382.02
S382.03
S382.04
S382.13
S382.14
S382.15
S382.16
S382.17
S380.01
S380.02
S380.03
S380.11
S380.12
S380.13
S380.14
S380.15
S380 Female | Male S382 [24 hours, 0 or 500ug/kg]
188
98
62
49
38
28
14
*
6
3
GAPDH
PPIA

## Slide 9
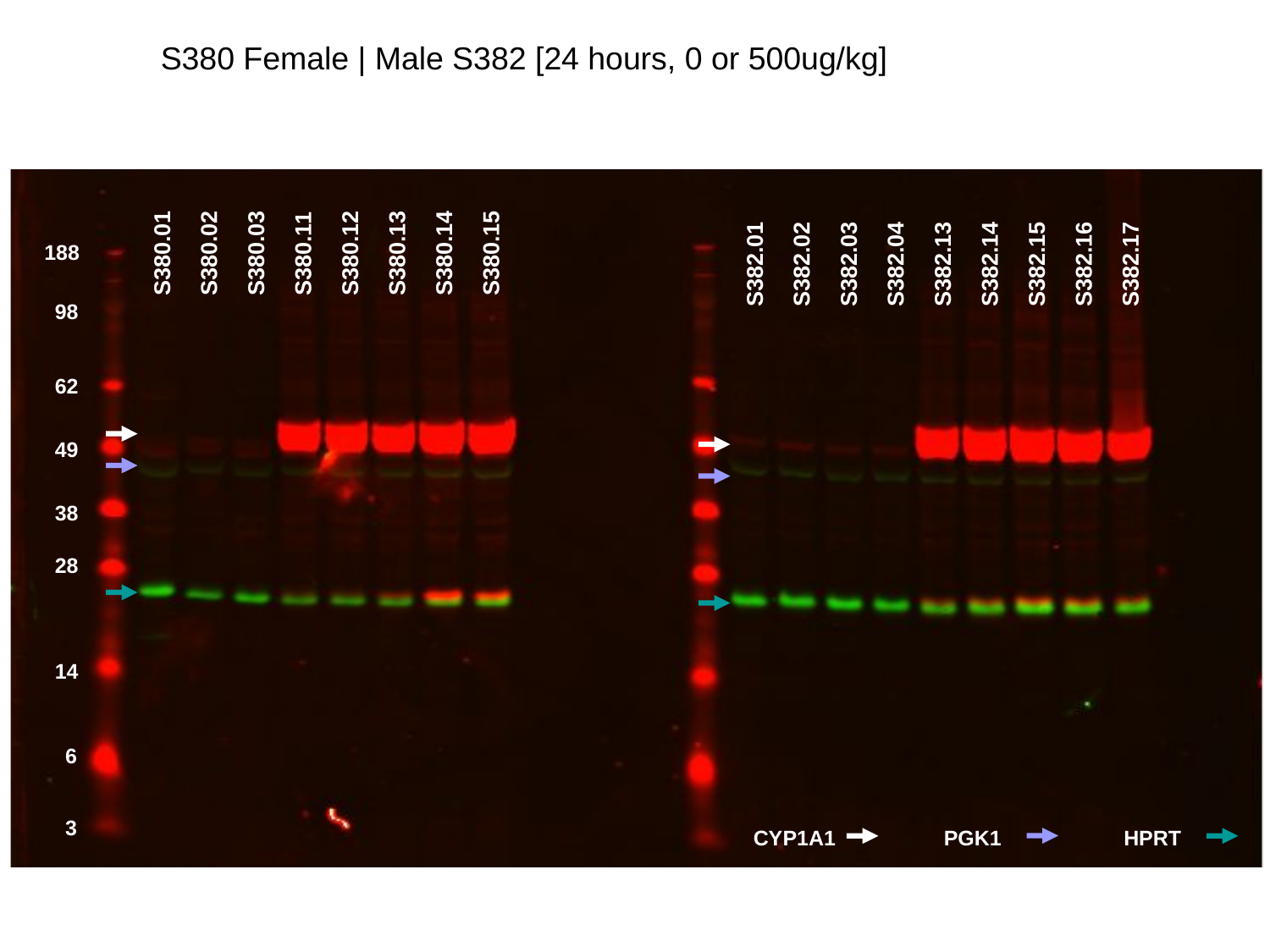

S382.01
S382.02
S382.03
S382.04
S382.13
S382.14
S382.15
S382.16
S382.17
S380 Female | Male S382 [24 hours, 0 or 500ug/kg]
S380.01
S380.02
S380.03
S380.11
S380.12
S380.13
S380.14
S380.15
188
98
62
49
38
28
14
6
3
CYP1A1
PGK1
HPRT

## Slide 10
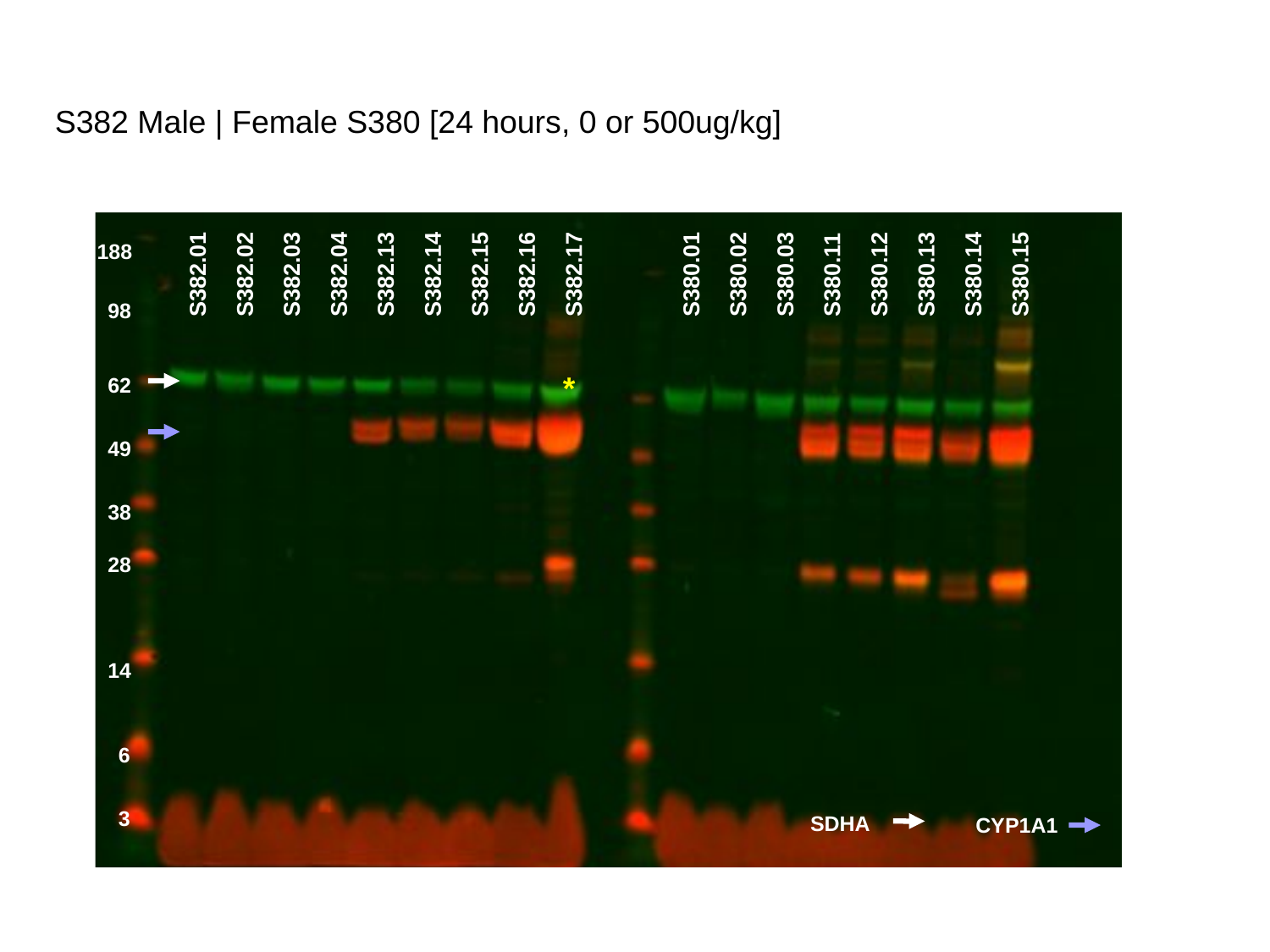

S382.01
S382.02
S382.03
S382.04
S382.13
S382.14
S382.15
S382.16
S382.17
S380.01
S380.02
S380.03
S380.11
S380.12
S380.13
S380.14
S380.15
S382 Male | Female S380 [24 hours, 0 or 500ug/kg]
188
98
*
62
49
38
28
14
6
3
SDHA
CYP1A1

## Slide 11
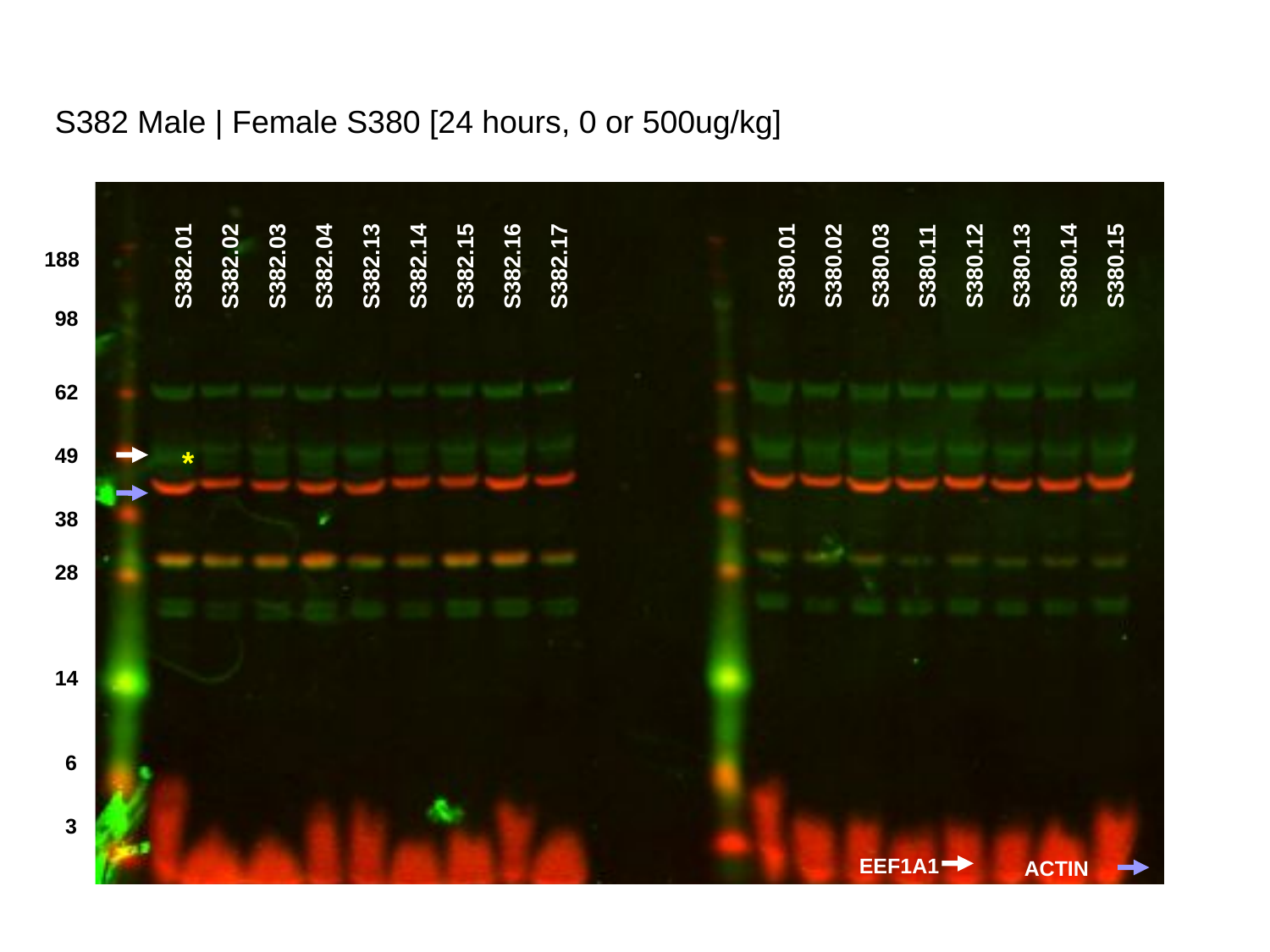

S382.01
S382.02
S382.03
S382.04
S382.13
S382.14
S382.15
S382.16
S382.17
S380.01
S380.02
S380.03
S380.11
S380.12
S380.13
S380.14
S380.15
S382 Male | Female S380 [24 hours, 0 or 500ug/kg]
188
98
62
*
49
38
28
14
6
3
EEF1A1
ACTIN

## Slide 12
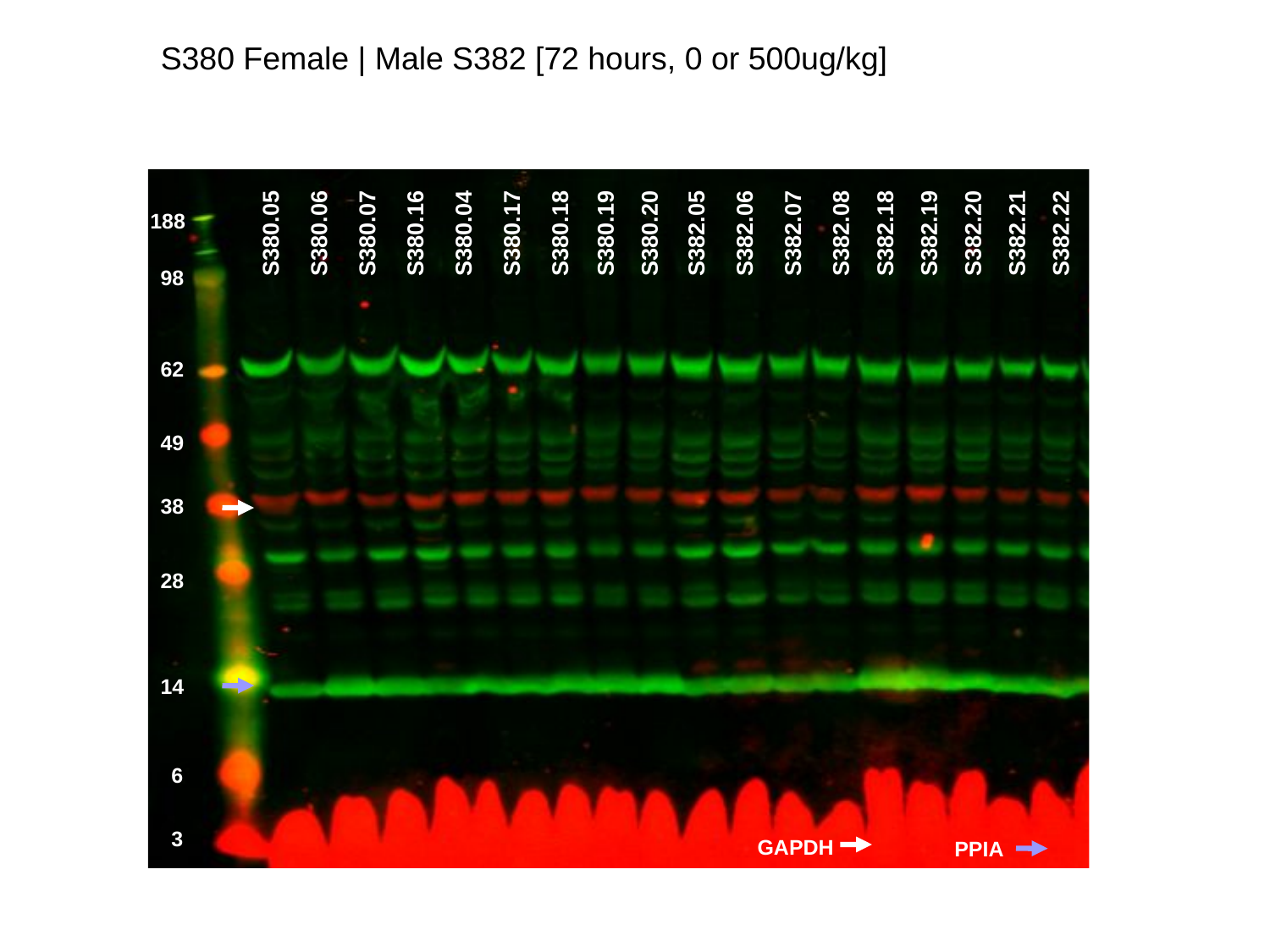

S380.05
S380.06
S380.07
S380.16
S380.04
S380.17
S380.18
S380.19
S380.20
S382.05
S382.06
S382.07
S382.08
S382.18
S382.19
S382.20
S382.21
S382.22
S380 Female | Male S382 [72 hours, 0 or 500ug/kg]
188
98
62
49
38
28
14
6
3
GAPDH
PPIA

## Slide 13
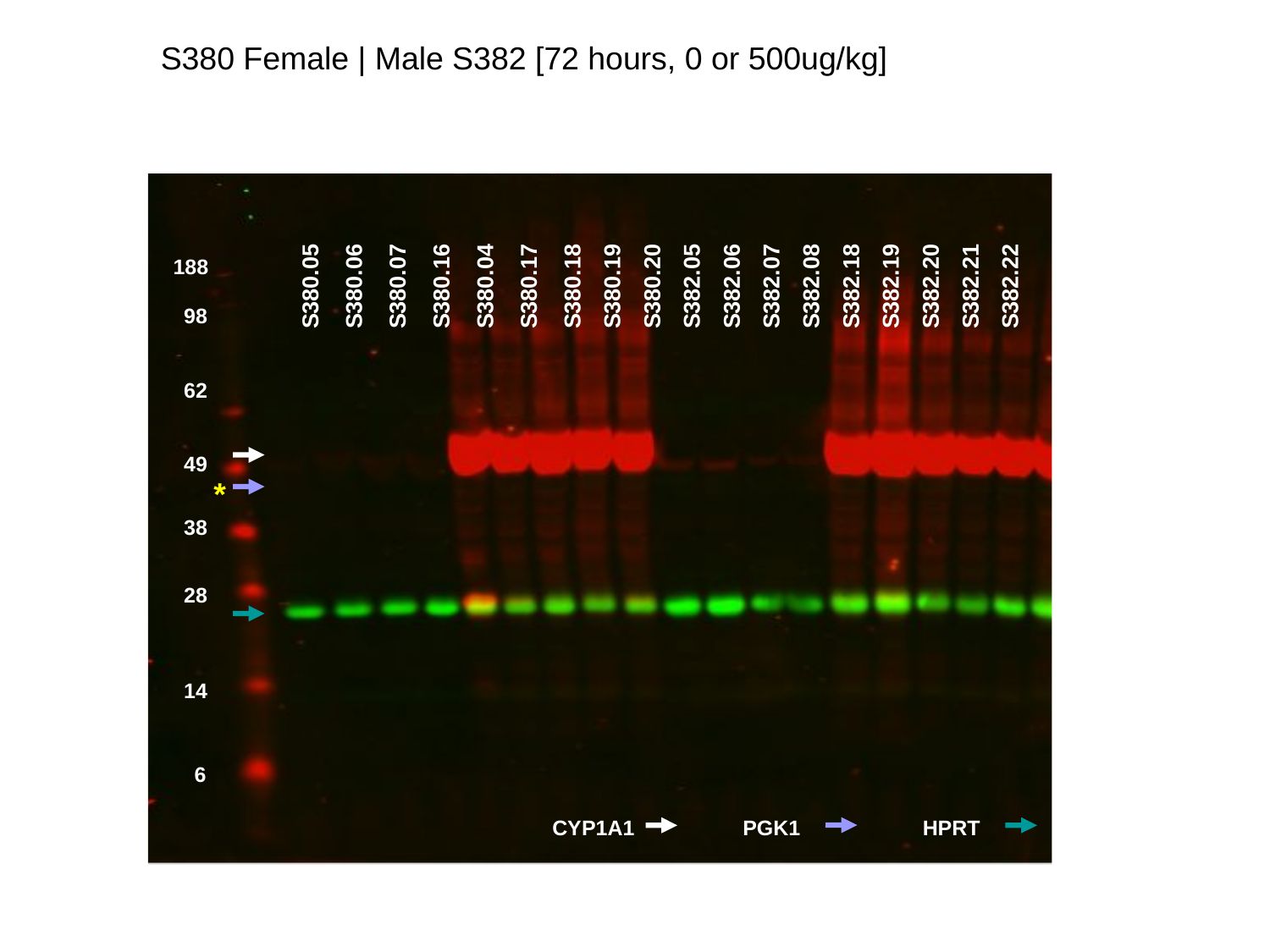

S380.05
S380.06
S380.07
S380.16
S380.04
S380.17
S380.18
S380.19
S380.20
S382.05
S382.06
S382.07
S382.08
S382.18
S382.19
S382.20
S382.21
S382.22
S380 Female | Male S382 [72 hours, 0 or 500ug/kg]
188
98
62
49
*
38
28
14
6
CYP1A1
PGK1
HPRT

## Slide 14
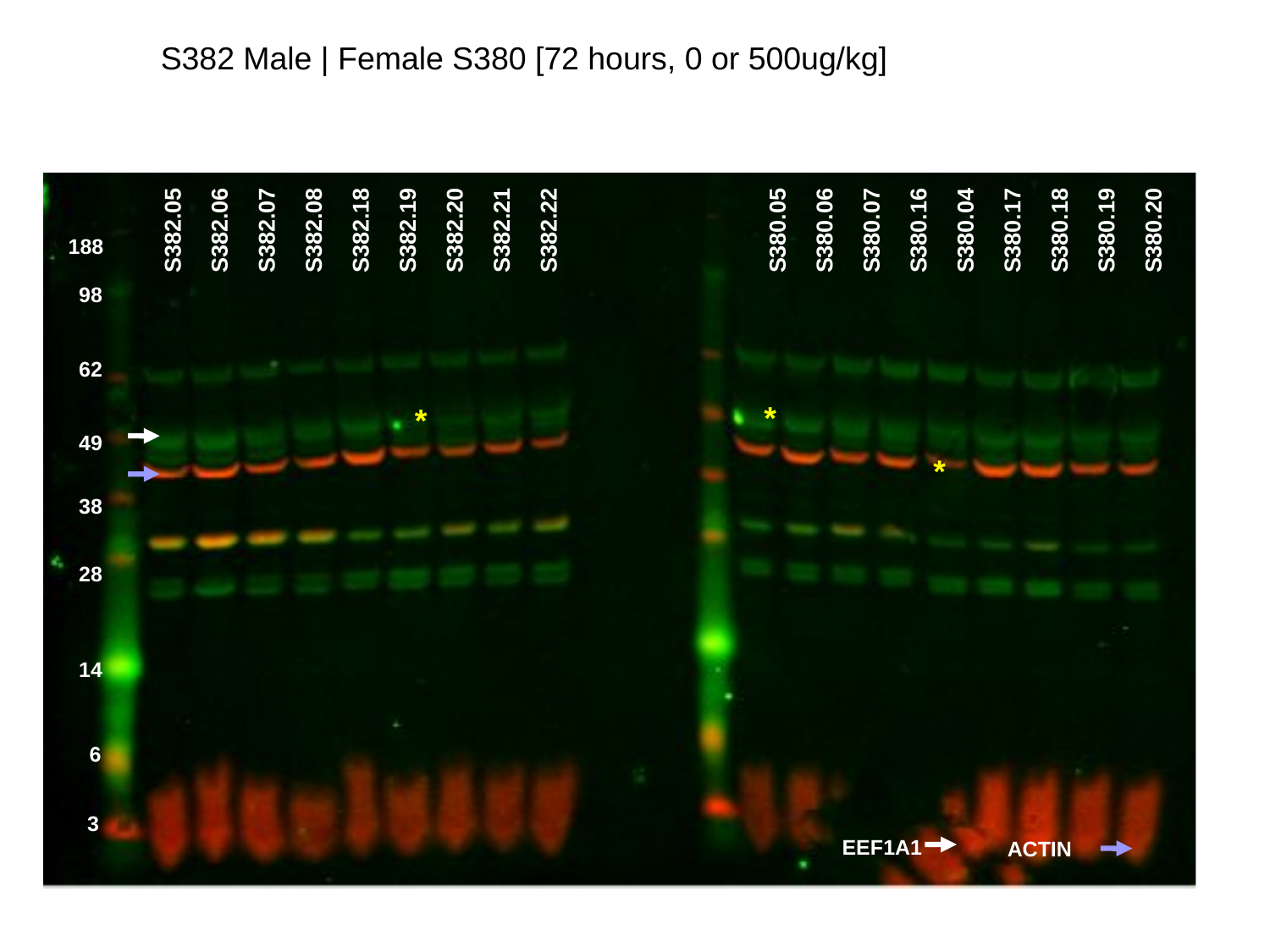

S382.05
S382.06
S382.07
S382.08
S382.18
S382.19
S382.20
S382.21
S382.22
S380.05
S380.06
S380.07
S380.16
S380.04
S380.17
S380.18
S380.19
S380.20
S382 Male | Female S380 [72 hours, 0 or 500ug/kg]
188
98
62
*
*
49
*
38
28
14
6
3
EEF1A1
ACTIN

## Slide 15
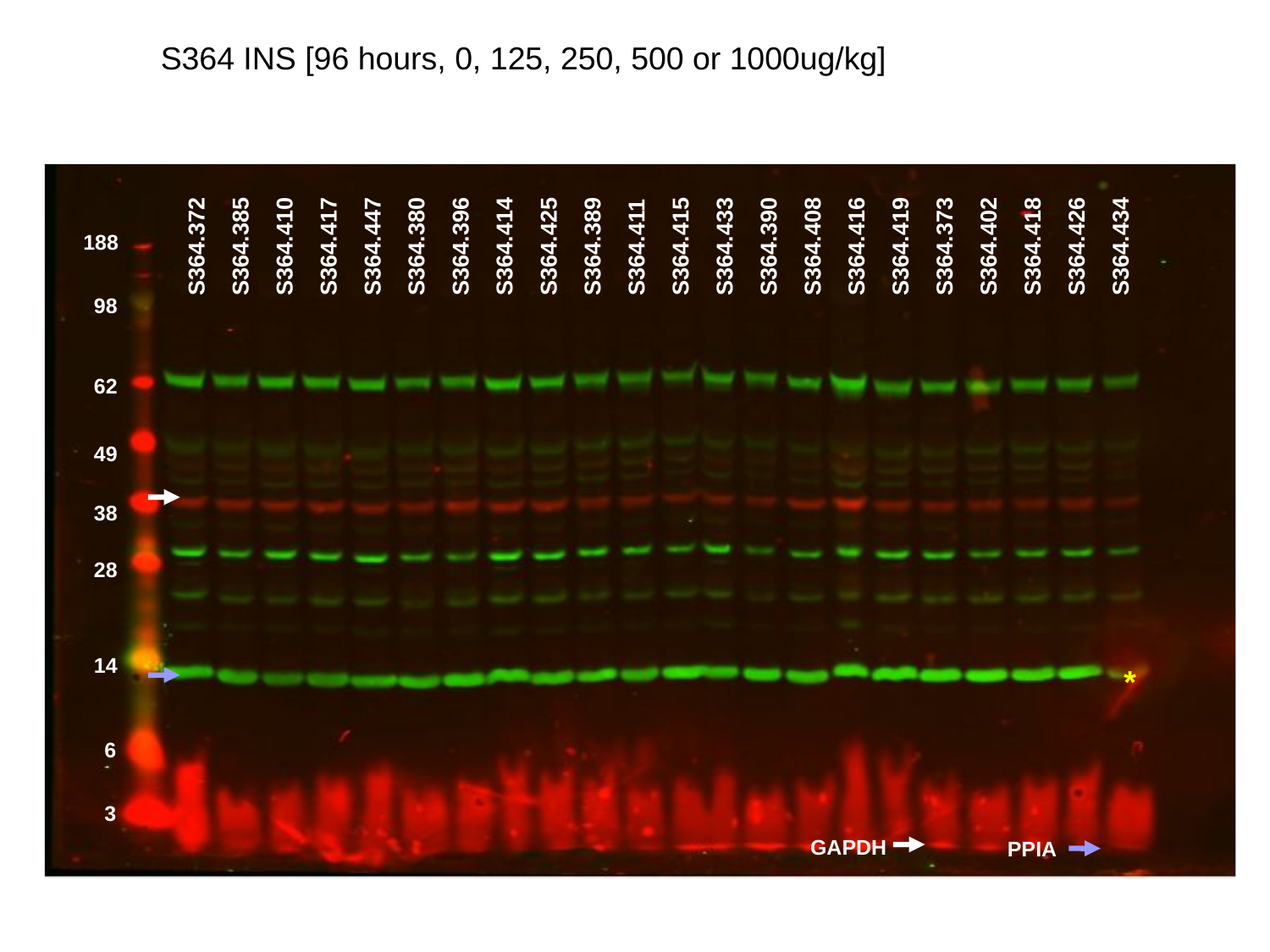

S364.372
S364.385
S364.410
S364.417
S364.447
S364.380
S364.396
S364.414
S364.425
S364.389
S364.411
S364.415
S364.433
S364.390
S364.408
S364.416
S364.419
S364.373
S364.402
S364.418
S364.426
S364.434
S364 INS [96 hours, 0, 125, 250, 500 or 1000ug/kg]
188
98
62
49
38
28
14
*
6
3
GAPDH
PPIA

## Slide 16
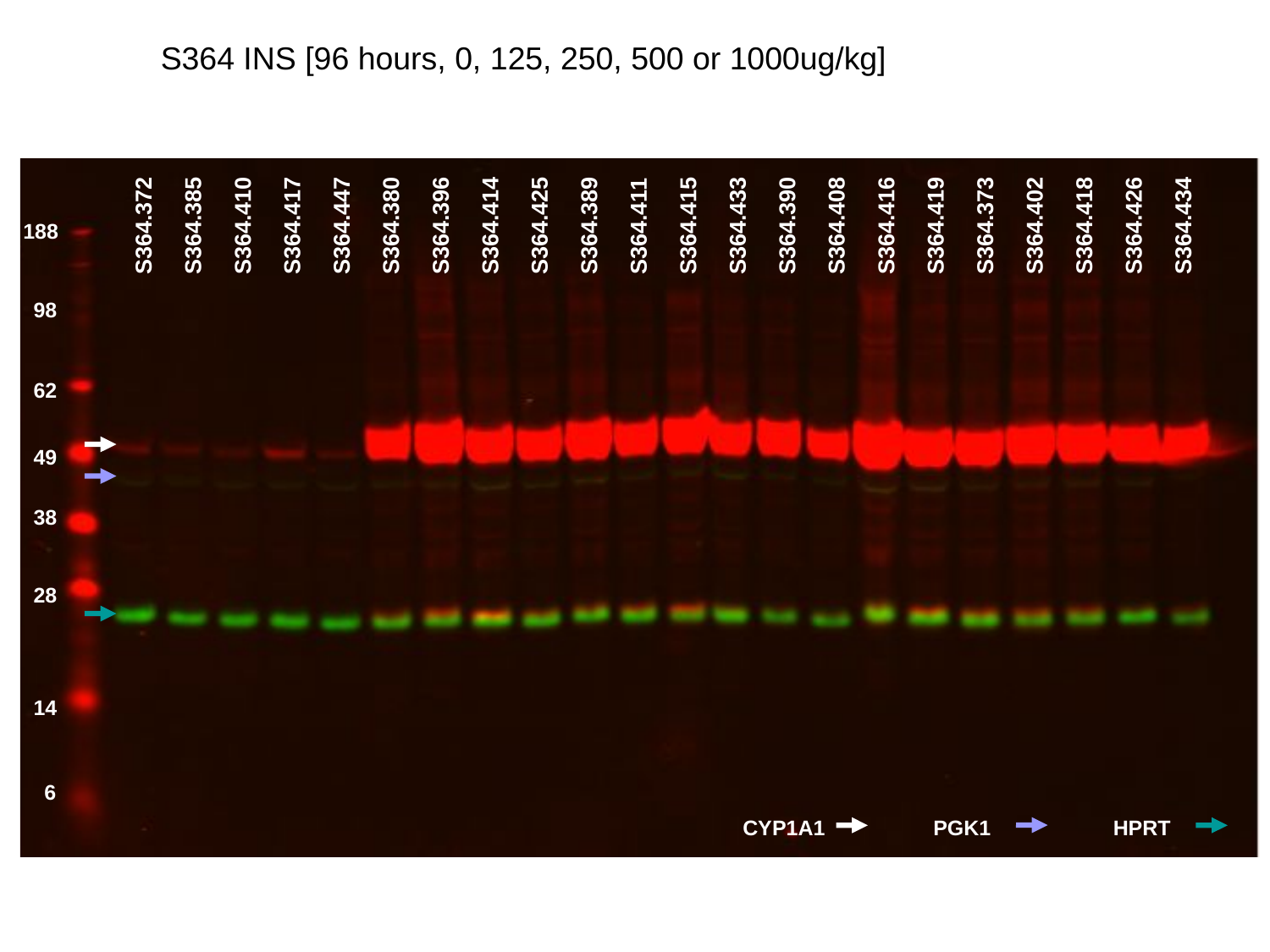

S364.372
S364.385
S364.410
S364.417
S364.447
S364.380
S364.396
S364.414
S364.425
S364.389
S364.411
S364.415
S364.433
S364.390
S364.408
S364.416
S364.419
S364.373
S364.402
S364.418
S364.426
S364.434
S364 INS [96 hours, 0, 125, 250, 500 or 1000ug/kg]
188
98
62
49
38
28
14
6
CYP1A1
PGK1
HPRT

## Slide 17
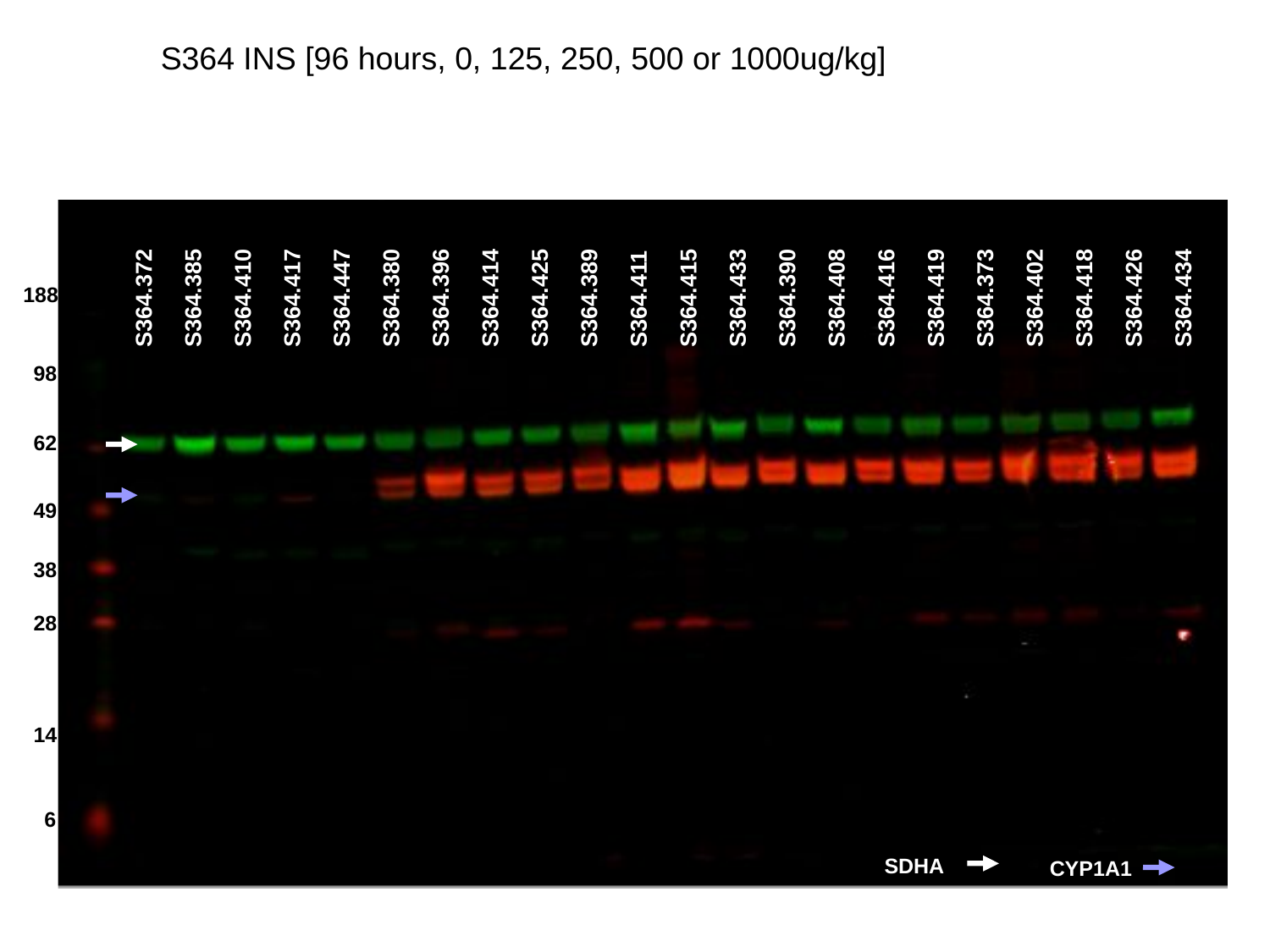

S364.372
S364.385
S364.410
S364.417
S364.447
S364.380
S364.396
S364.414
S364.425
S364.389
S364.411
S364.415
S364.433
S364.390
S364.408
S364.416
S364.419
S364.373
S364.402
S364.418
S364.426
S364.434
S364 INS [96 hours, 0, 125, 250, 500 or 1000ug/kg]
188
98
62
49
38
28
14
6
SDHA
CYP1A1

## Slide 18
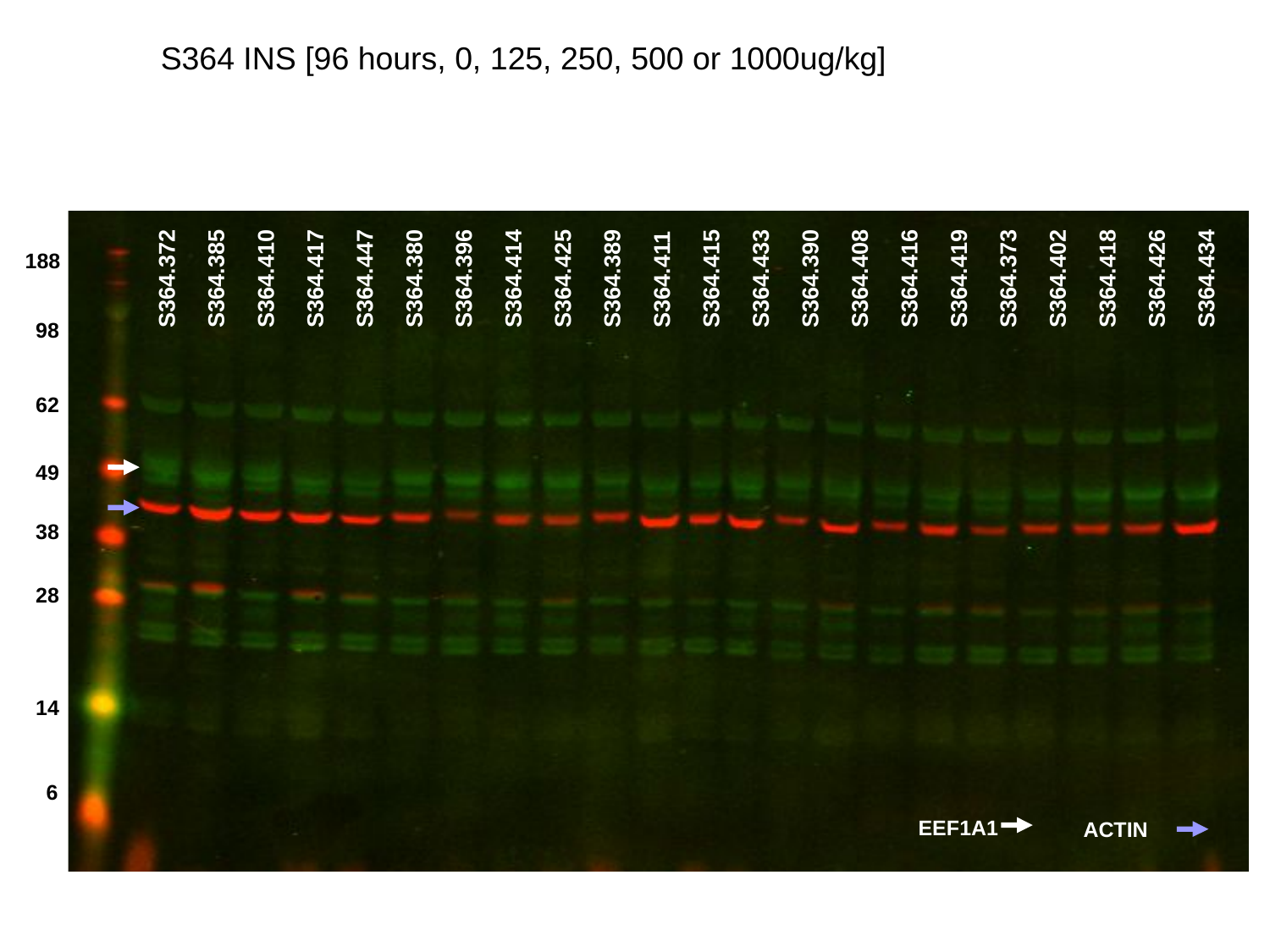

S364.372
S364.385
S364.410
S364.417
S364.447
S364.380
S364.396
S364.414
S364.425
S364.389
S364.411
S364.415
S364.433
S364.390
S364.408
S364.416
S364.419
S364.373
S364.402
S364.418
S364.426
S364.434
S364 INS [96 hours, 0, 125, 250, 500 or 1000ug/kg]
188
98
62
49
38
28
14
6
EEF1A1
ACTIN

## Slide 19
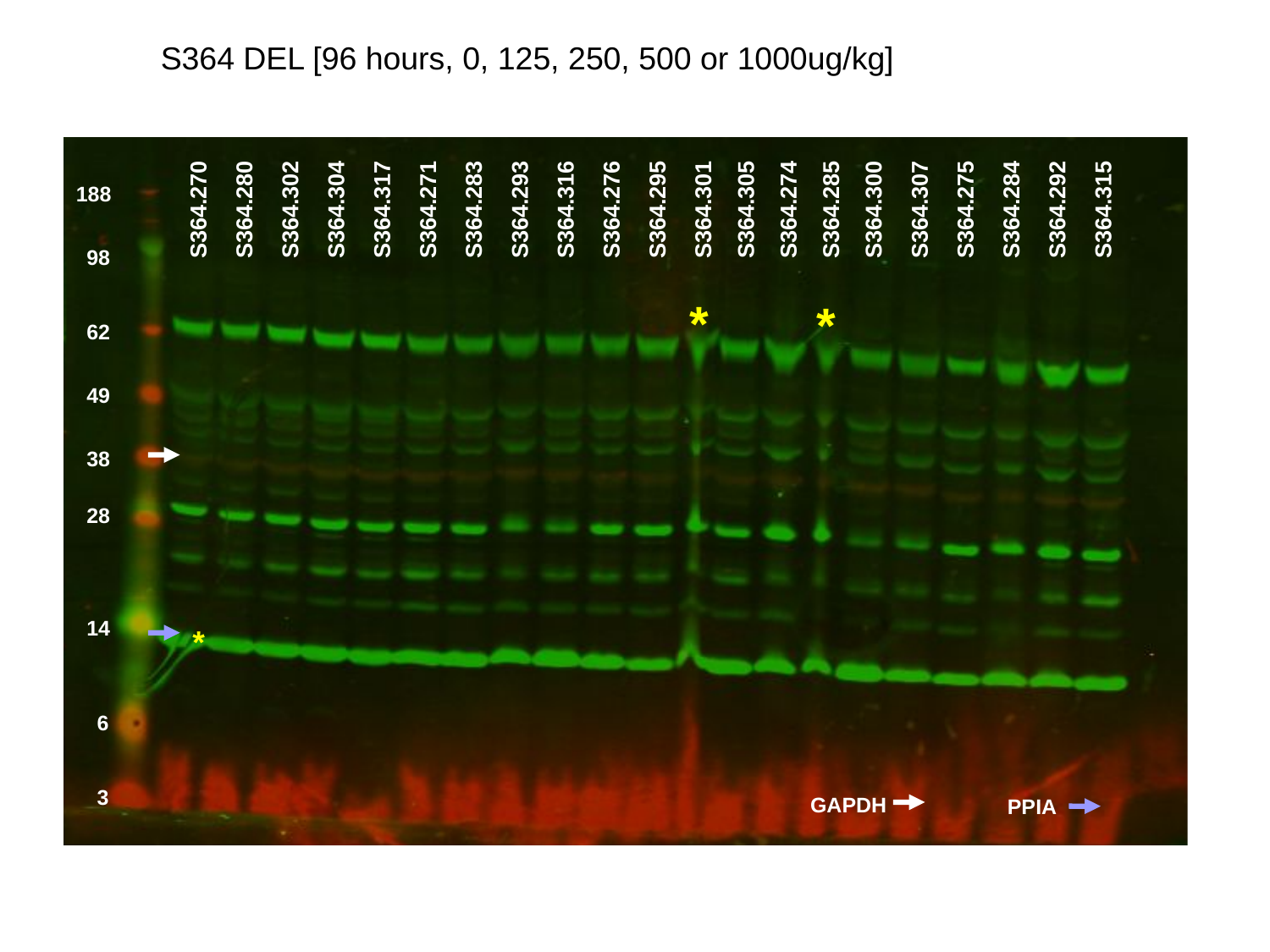

S364.270
S364.280
S364.302
S364.304
S364.317
S364.271
S364.283
S364.293
S364.316
S364.276
S364.295
S364.301
S364.305
S364.274
S364.285
S364.300
S364.307
S364.275
S364.284
S364.292
S364.315
S364 DEL [96 hours, 0, 125, 250, 500 or 1000ug/kg]
188
98
*
*
62
49
38
28
14
*
6
3
GAPDH
PPIA

## Slide 20
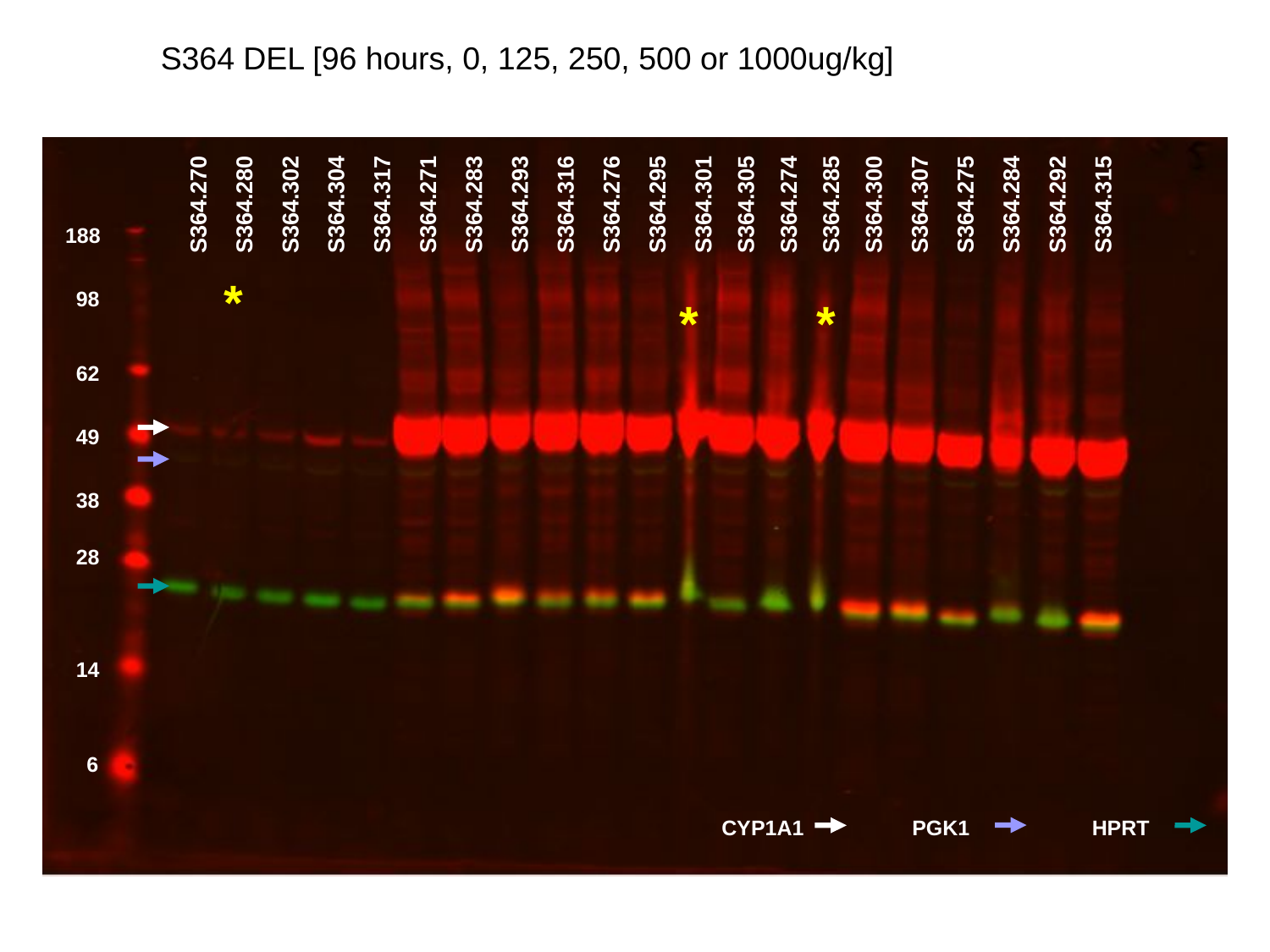

S364.270
S364.280
S364.302
S364.304
S364.317
S364.271
S364.283
S364.293
S364.316
S364.276
S364.295
S364.301
S364.305
S364.274
S364.285
S364.300
S364.307
S364.275
S364.284
S364.292
S364.315
S364 DEL [96 hours, 0, 125, 250, 500 or 1000ug/kg]
188
*
98
*
*
62
49
38
28
14
6
CYP1A1
PGK1
HPRT

## Slide 21
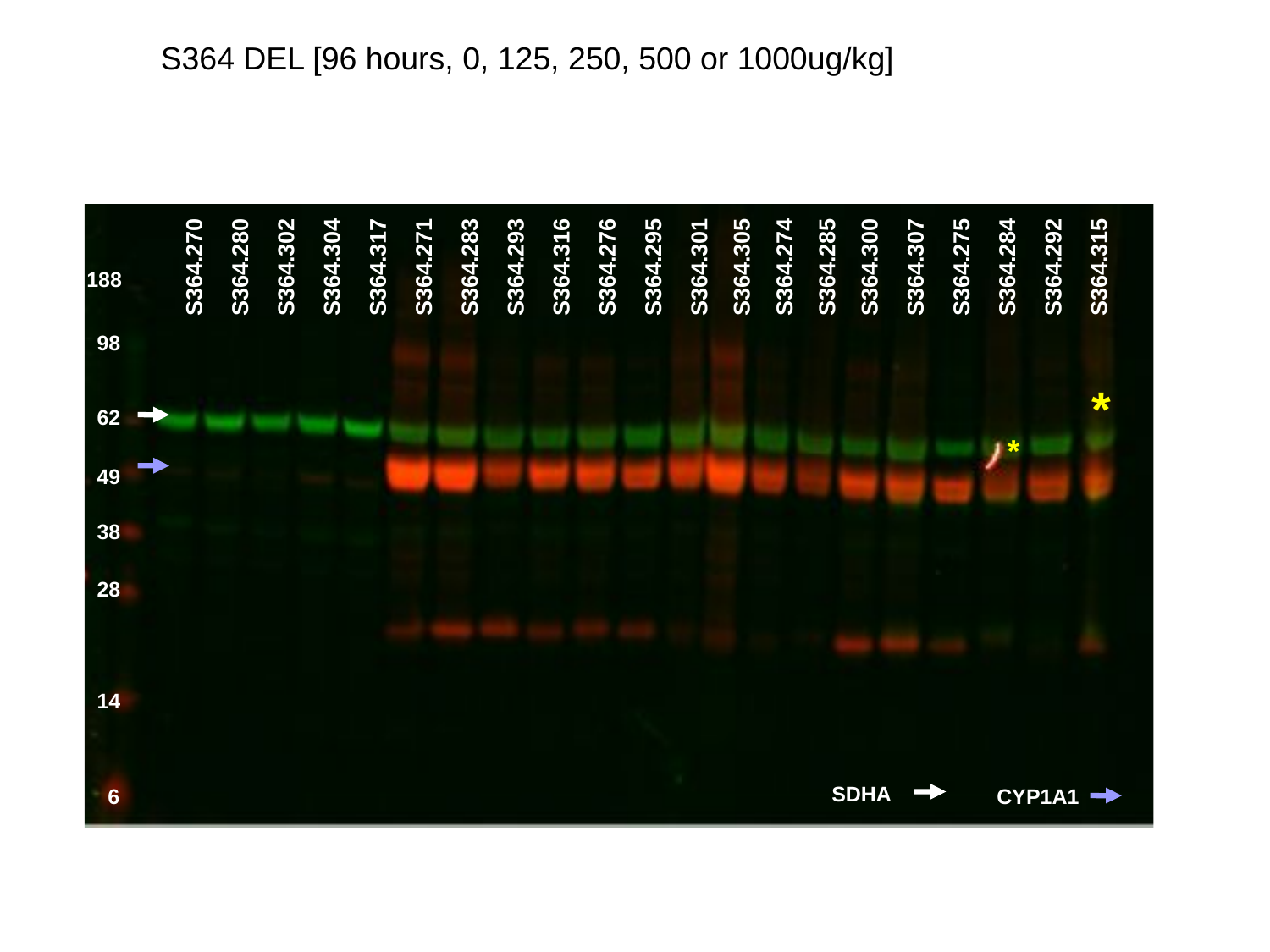

S364.270
S364.280
S364.302
S364.304
S364.317
S364.271
S364.283
S364.293
S364.316
S364.276
S364.295
S364.301
S364.305
S364.274
S364.285
S364.300
S364.307
S364.275
S364.284
S364.292
S364.315
S364 DEL [96 hours, 0, 125, 250, 500 or 1000ug/kg]
188
98
*
62
*
49
38
28
14
SDHA
6
CYP1A1
3

## Slide 22
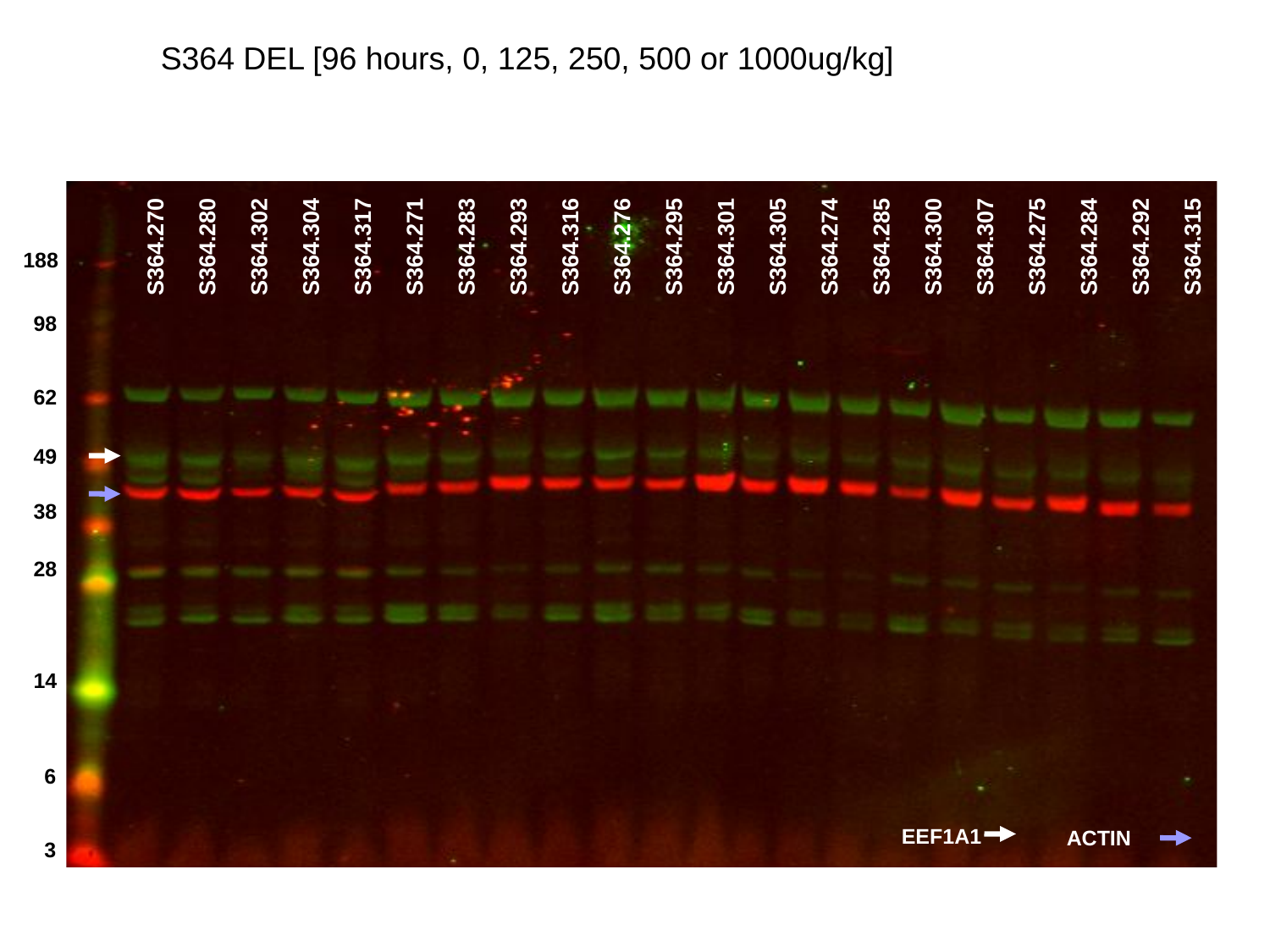

S364.270
S364.280
S364.302
S364.304
S364.317
S364.271
S364.283
S364.293
S364.316
S364.276
S364.295
S364.301
S364.305
S364.274
S364.285
S364.300
S364.307
S364.275
S364.284
S364.292
S364.315
S364 DEL [96 hours, 0, 125, 250, 500 or 1000ug/kg]
188
98
62
49
38
28
14
6
EEF1A1
ACTIN
3

## Slide 23
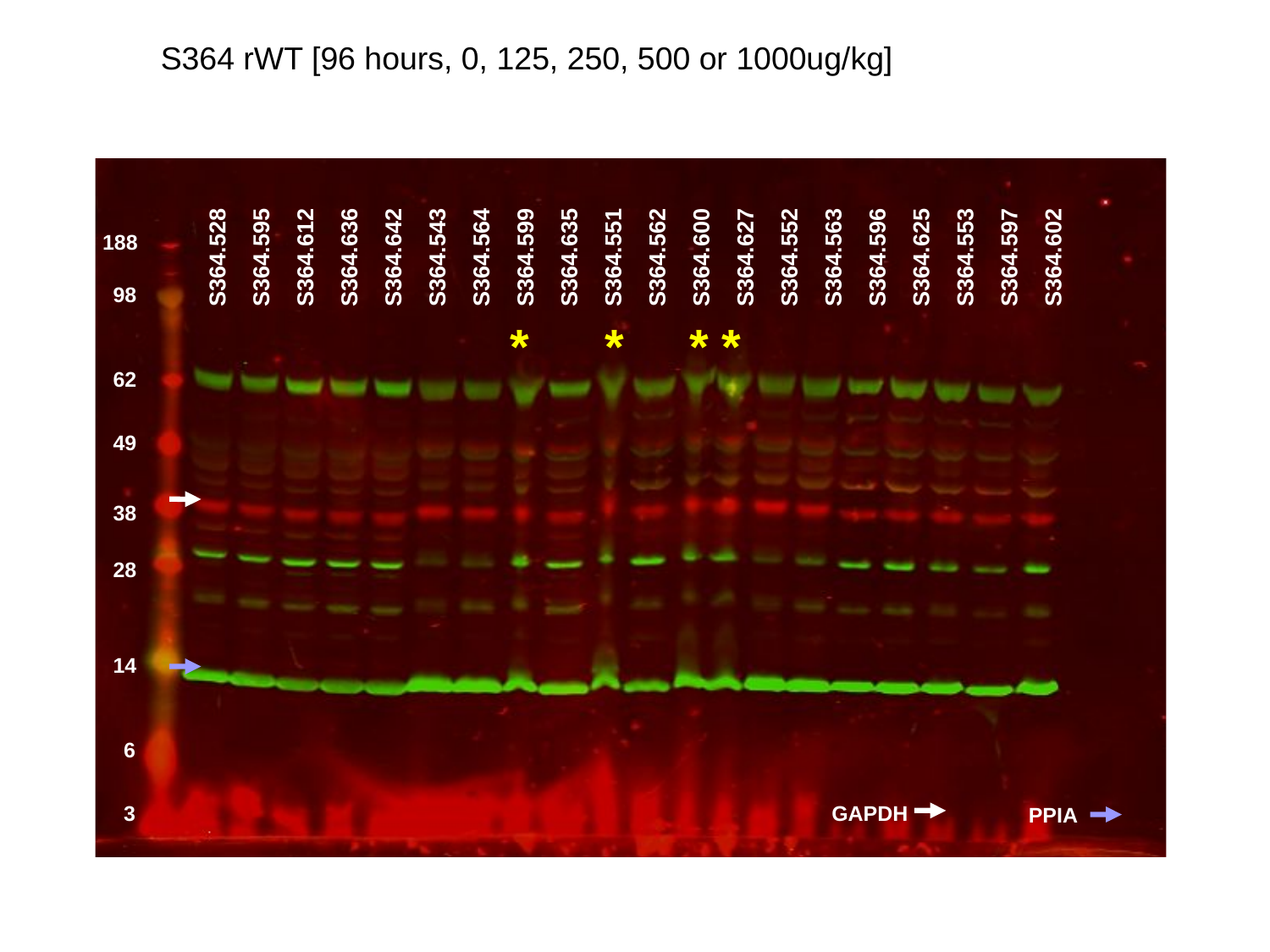

S364.528
S364.595
S364.612
S364.636
S364.642
S364.543
S364.564
S364.599
S364.635
S364.551
S364.562
S364.600
S364.627
S364.552
S364.563
S364.596
S364.625
S364.553
S364.597
S364.602
S364 rWT [96 hours, 0, 125, 250, 500 or 1000ug/kg]
188
98
*
*
*
*
62
49
38
28
14
6
3
GAPDH
PPIA

## Slide 24
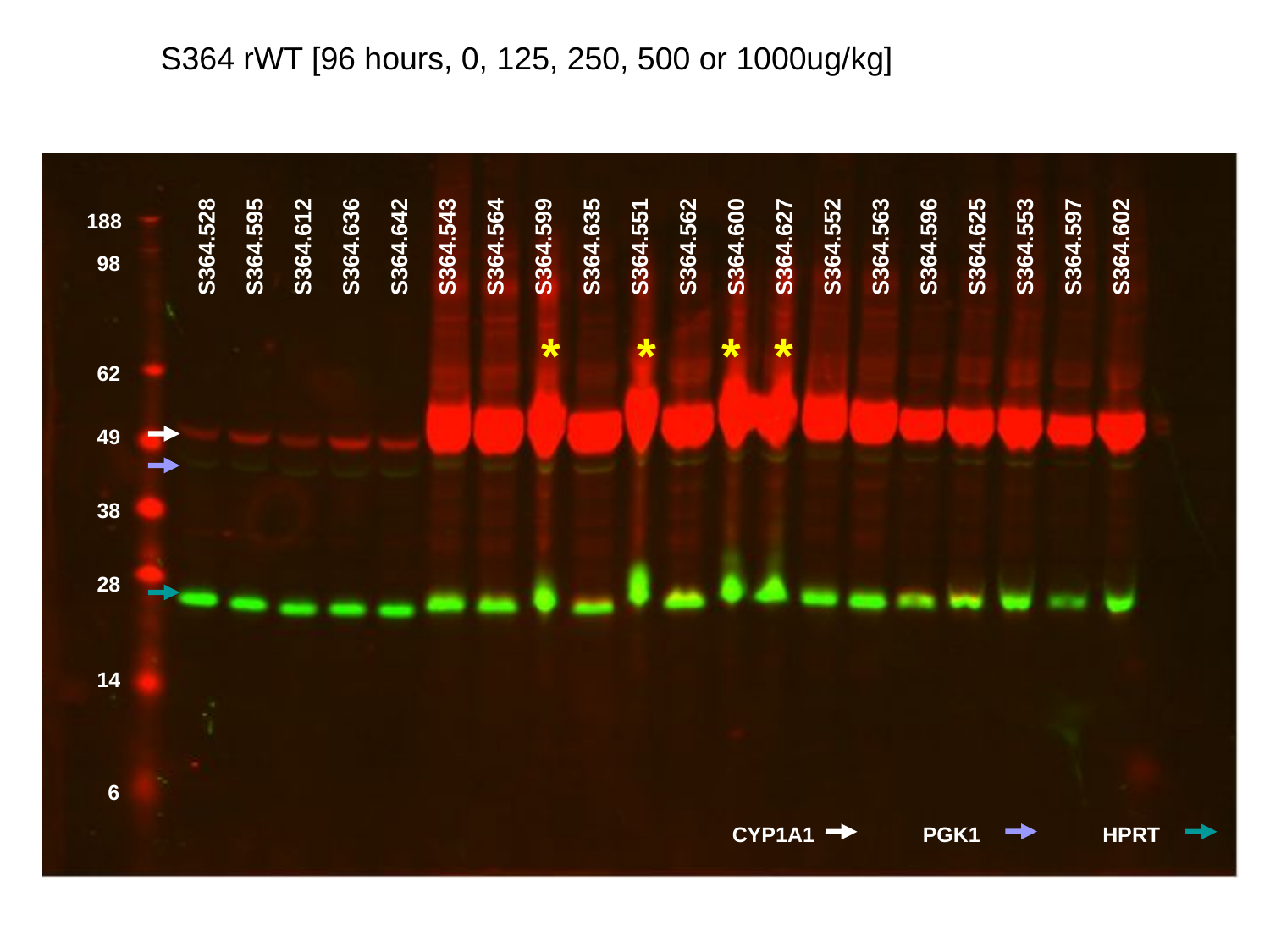

S364.528
S364.595
S364.612
S364.636
S364.642
S364.543
S364.564
S364.599
S364.635
S364.551
S364.562
S364.600
S364.627
S364.552
S364.563
S364.596
S364.625
S364.553
S364.597
S364.602
S364 rWT [96 hours, 0, 125, 250, 500 or 1000ug/kg]
188
98
*
*
*
*
62
49
38
28
14
6
CYP1A1
PGK1
HPRT

## Slide 25
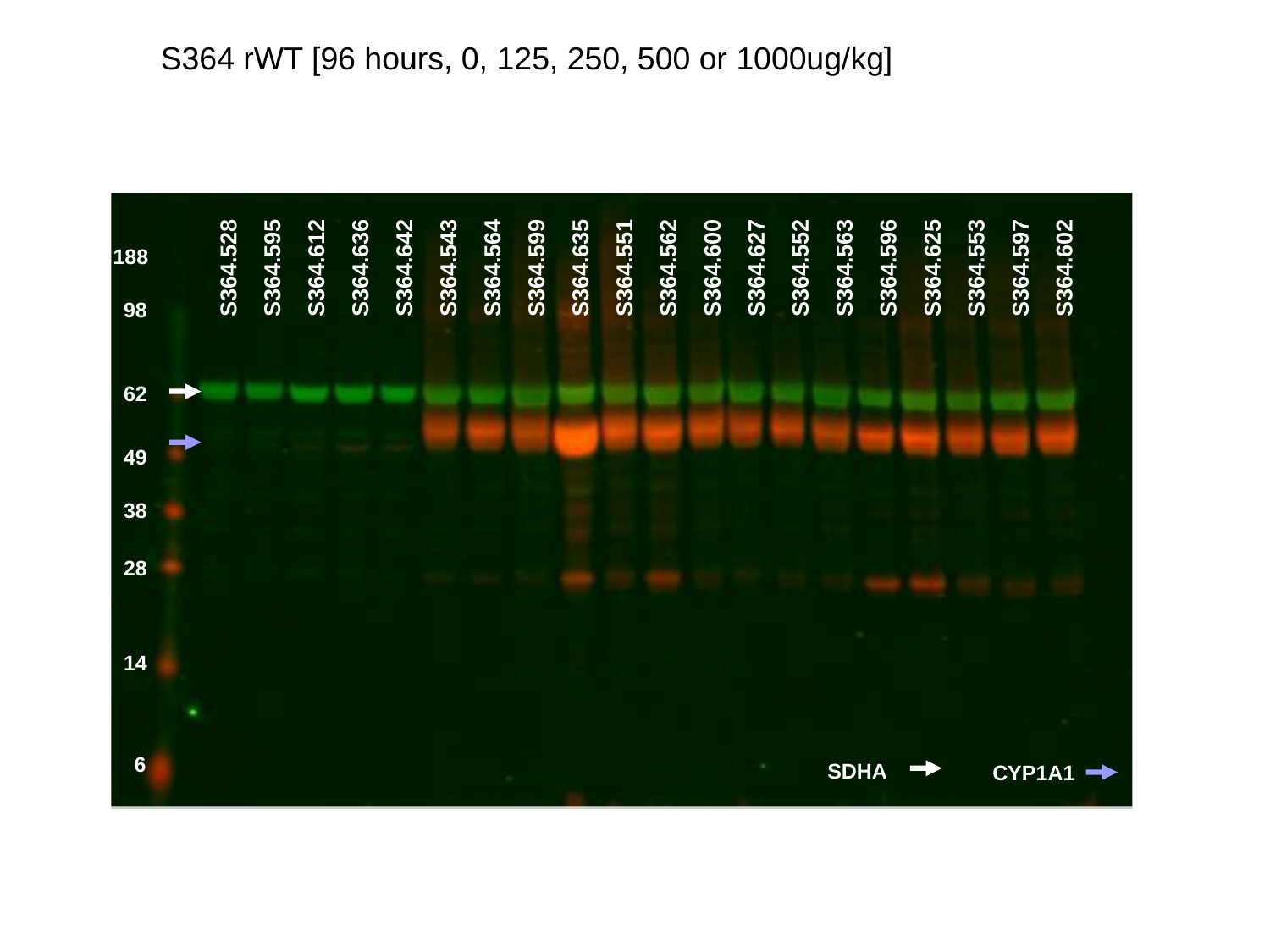

S364.528
S364.595
S364.612
S364.636
S364.642
S364.543
S364.564
S364.599
S364.635
S364.551
S364.562
S364.600
S364.627
S364.552
S364.563
S364.596
S364.625
S364.553
S364.597
S364.602
S364 rWT [96 hours, 0, 125, 250, 500 or 1000ug/kg]
188
98
62
49
38
28
14
6
SDHA
CYP1A1
3

## Slide 26
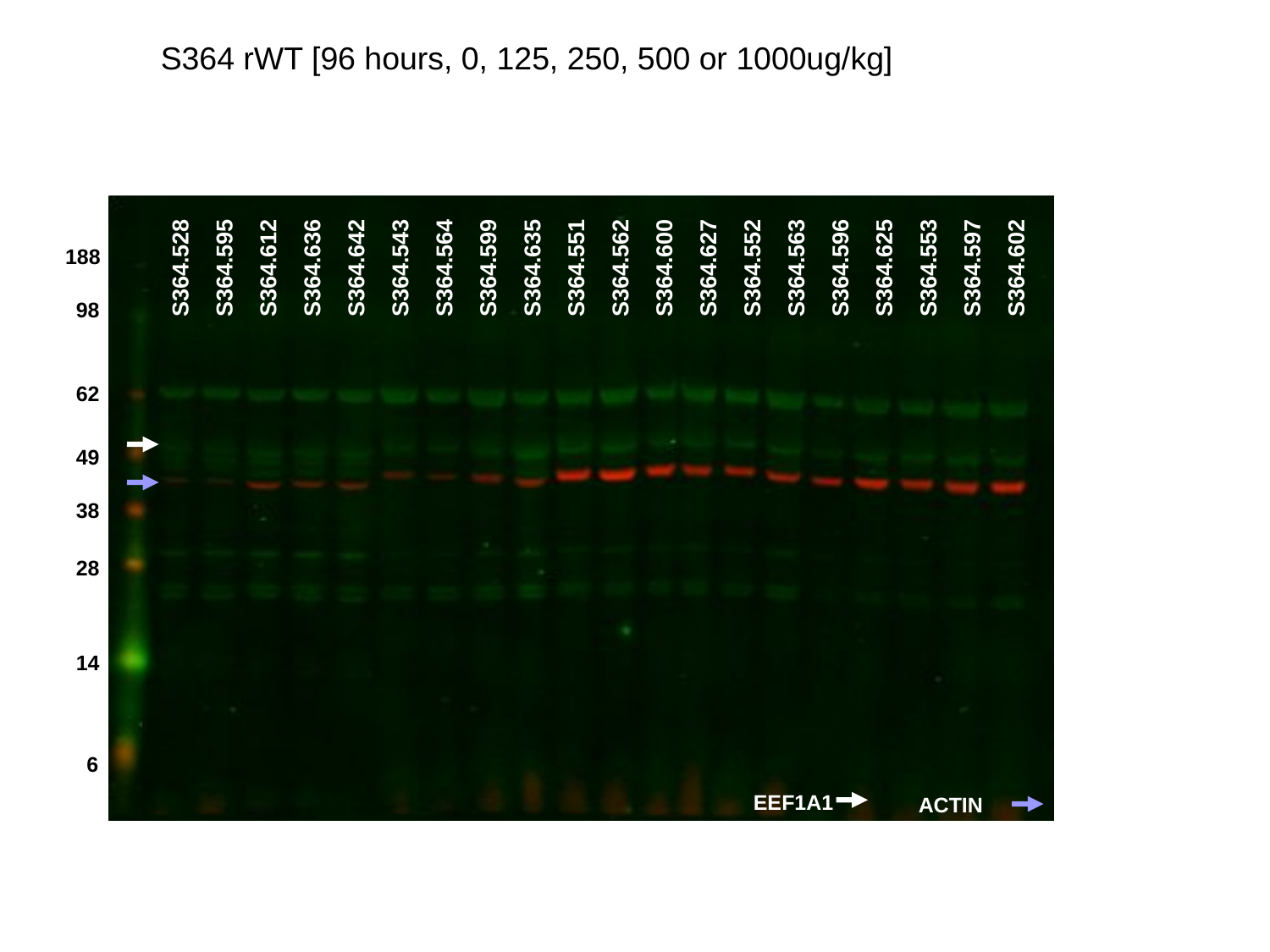

S364.528
S364.595
S364.612
S364.636
S364.642
S364.543
S364.564
S364.599
S364.635
S364.551
S364.562
S364.600
S364.627
S364.552
S364.563
S364.596
S364.625
S364.553
S364.597
S364.602
S364 rWT [96 hours, 0, 125, 250, 500 or 1000ug/kg]
188
98
62
49
38
28
14
6
EEF1A1
ACTIN

## Slide 27
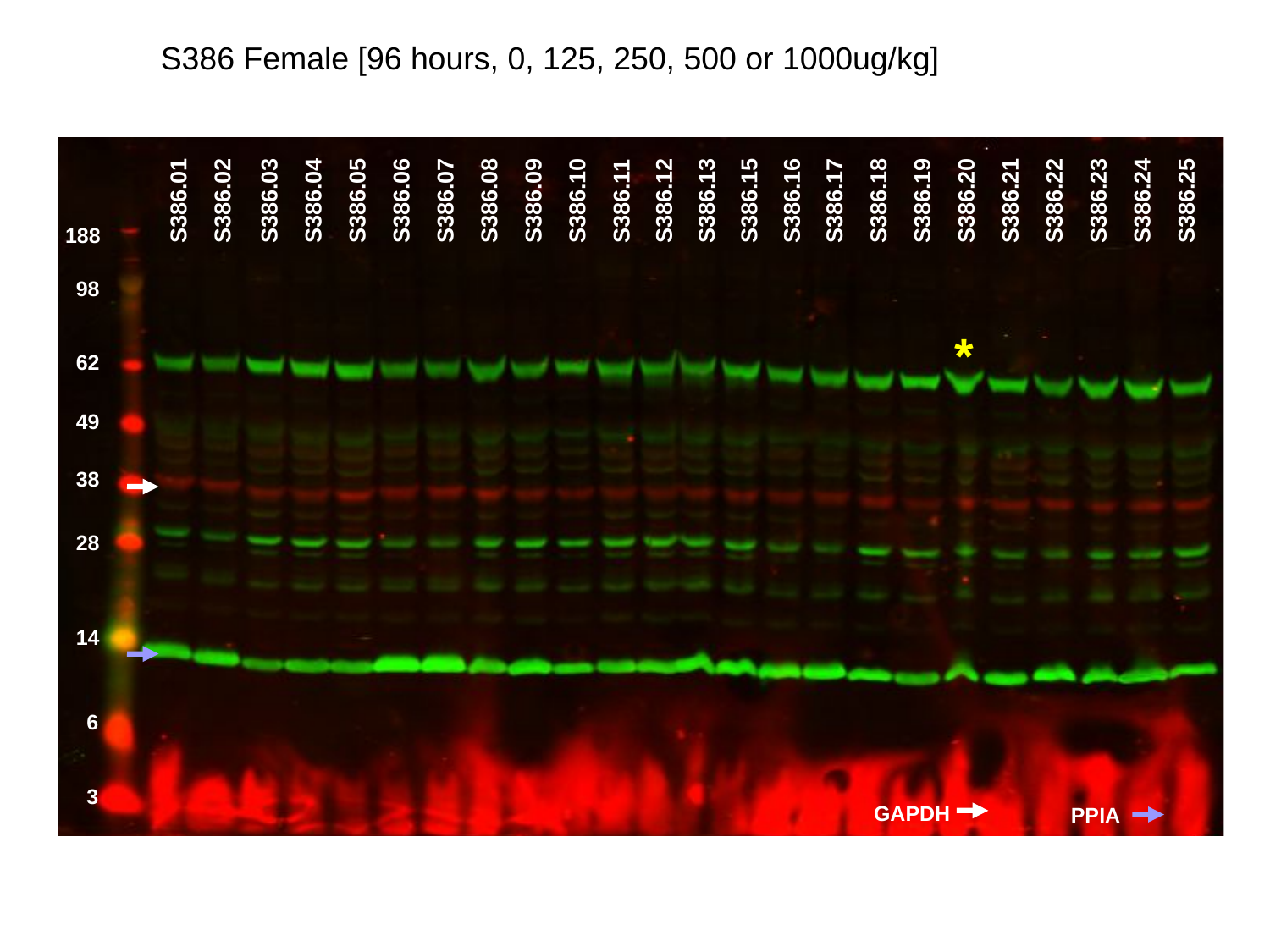

S386.01
S386.02
S386.03
S386.04
S386.05
S386.06
S386.07
S386.08
S386.09
S386.10
S386.11
S386.12
S386.13
S386.15
S386.16
S386.17
S386.18
S386.19
S386.20
S386.21
S386.22
S386.23
S386.24
S386.25
S386 Female [96 hours, 0, 125, 250, 500 or 1000ug/kg]
188
98
*
62
49
38
28
14
6
3
GAPDH
PPIA

## Slide 28
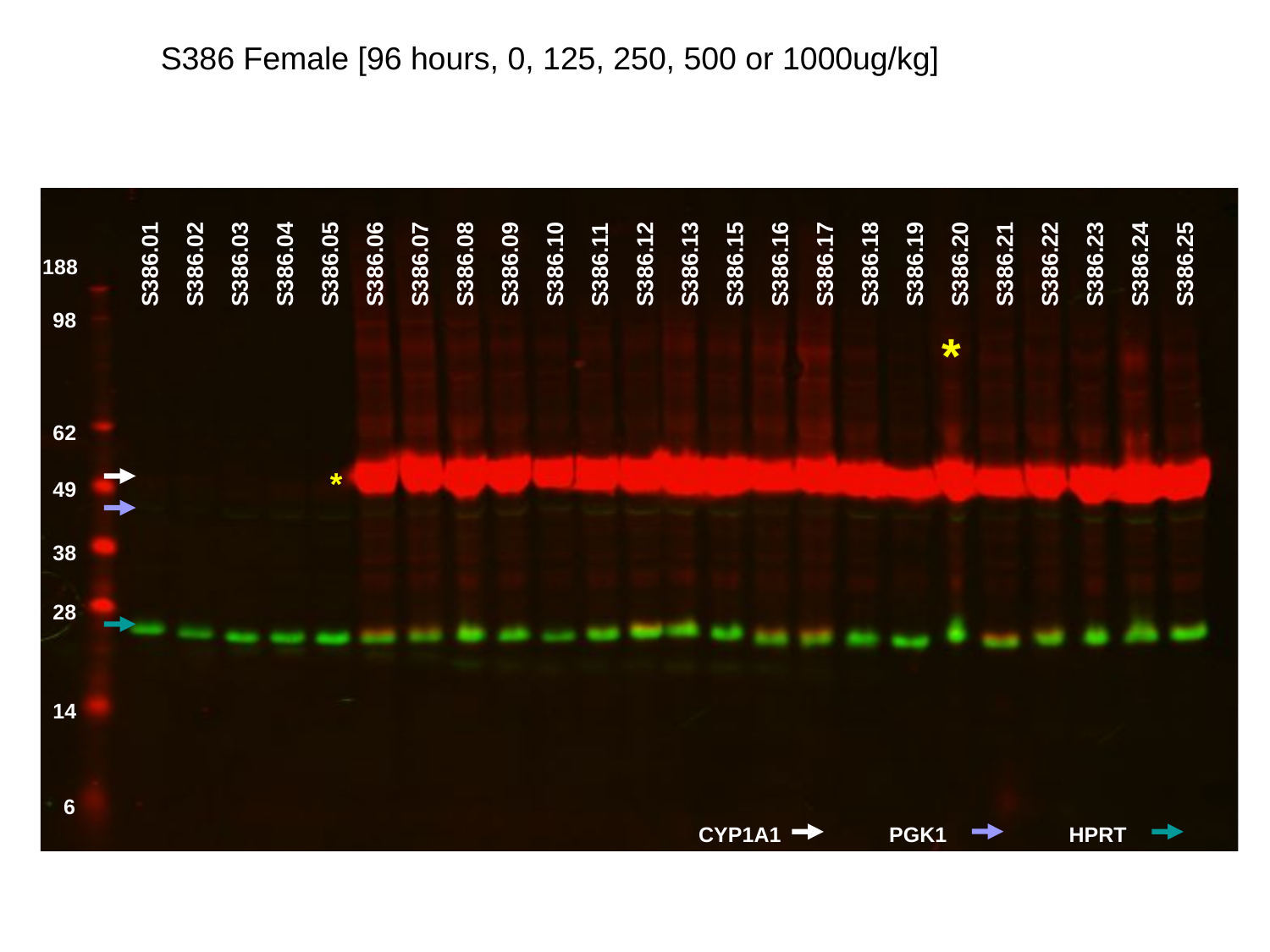

S386.01
S386.02
S386.03
S386.04
S386.05
S386.06
S386.07
S386.08
S386.09
S386.10
S386.11
S386.12
S386.13
S386.15
S386.16
S386.17
S386.18
S386.19
S386.20
S386.21
S386.22
S386.23
S386.24
S386.25
S386 Female [96 hours, 0, 125, 250, 500 or 1000ug/kg]
188
98
*
62
*
49
38
28
14
6
CYP1A1
PGK1
HPRT

## Slide 29
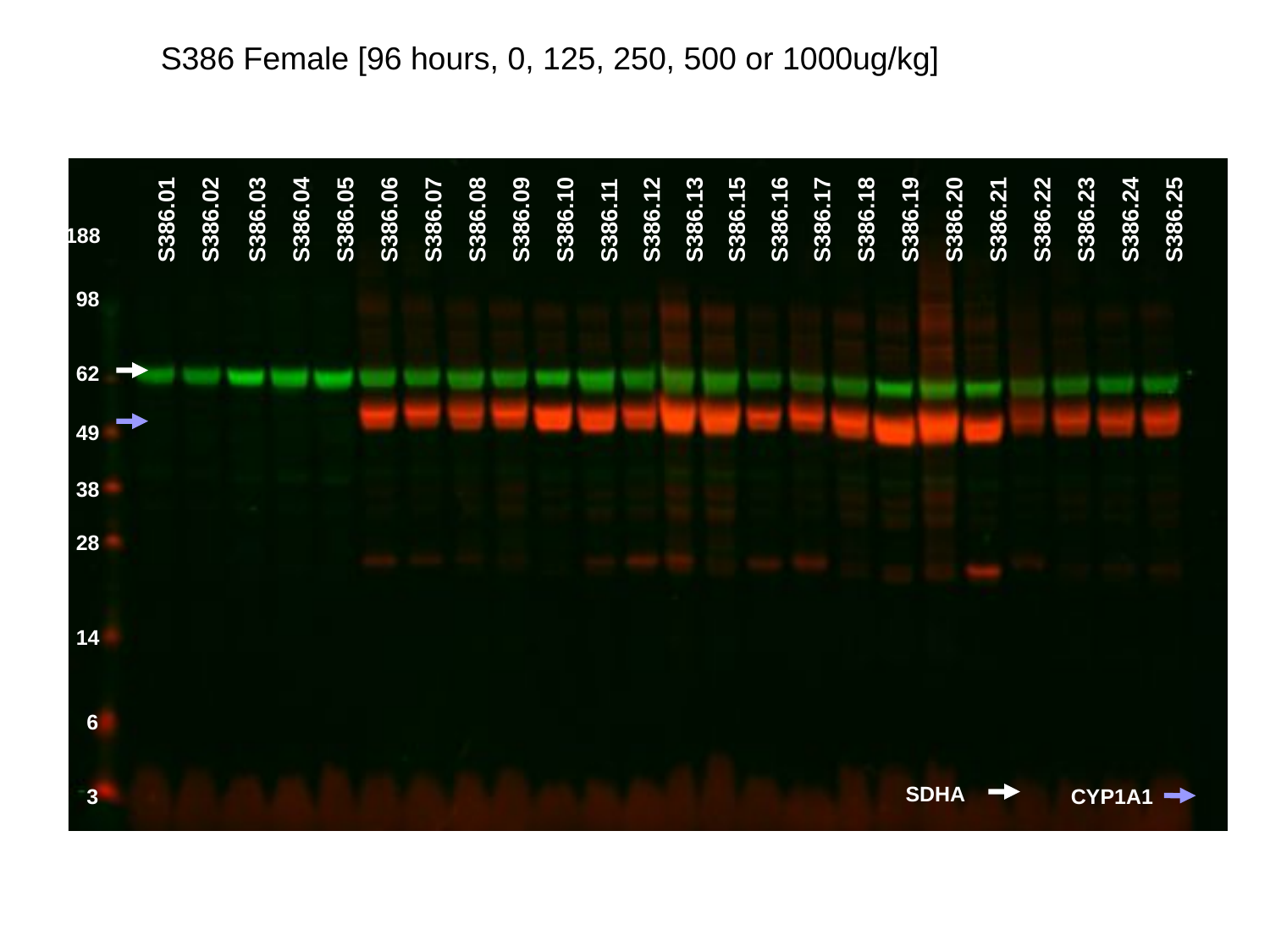

S386.01
S386.02
S386.03
S386.04
S386.05
S386.06
S386.07
S386.08
S386.09
S386.10
S386.11
S386.12
S386.13
S386.15
S386.16
S386.17
S386.18
S386.19
S386.20
S386.21
S386.22
S386.23
S386.24
S386.25
S386 Female [96 hours, 0, 125, 250, 500 or 1000ug/kg]
188
98
62
49
38
28
14
6
SDHA
3
CYP1A1

## Slide 30
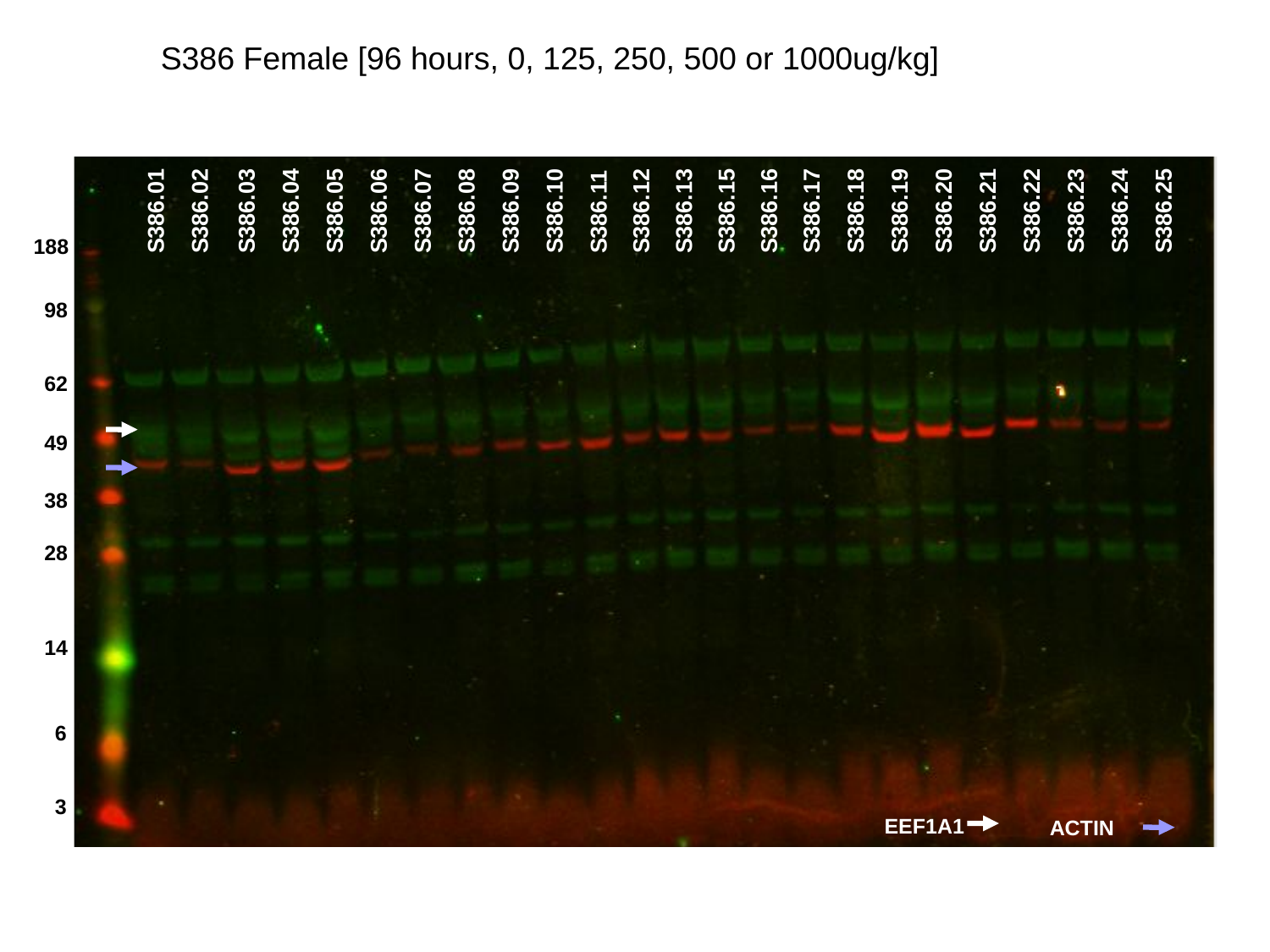

S386.01
S386.02
S386.03
S386.04
S386.05
S386.06
S386.07
S386.08
S386.09
S386.10
S386.11
S386.12
S386.13
S386.15
S386.16
S386.17
S386.18
S386.19
S386.20
S386.21
S386.22
S386.23
S386.24
S386.25
S386 Female [96 hours, 0, 125, 250, 500 or 1000ug/kg]
188
98
62
49
38
28
14
6
3
EEF1A1
ACTIN

## Slide 31
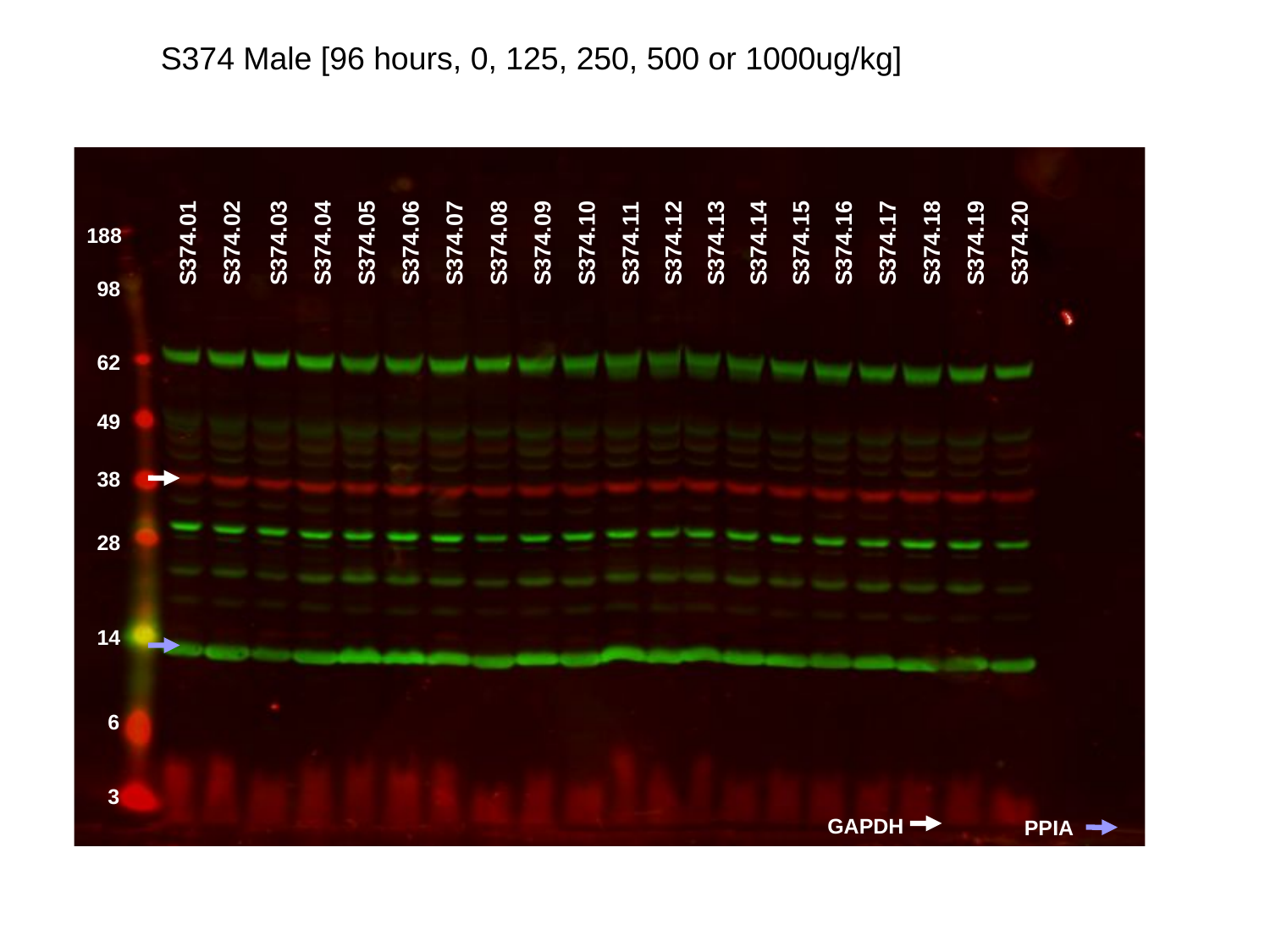

S374.01
S374.02
S374.03
S374.04
S374.05
S374.06
S374.07
S374.08
S374.09
S374.10
S374.11
S374.12
S374.13
S374.14
S374.15
S374.16
S374.17
S374.18
S374.19
S374.20
S374 Male [96 hours, 0, 125, 250, 500 or 1000ug/kg]
188
98
62
49
38
28
14
6
3
GAPDH
PPIA

## Slide 32
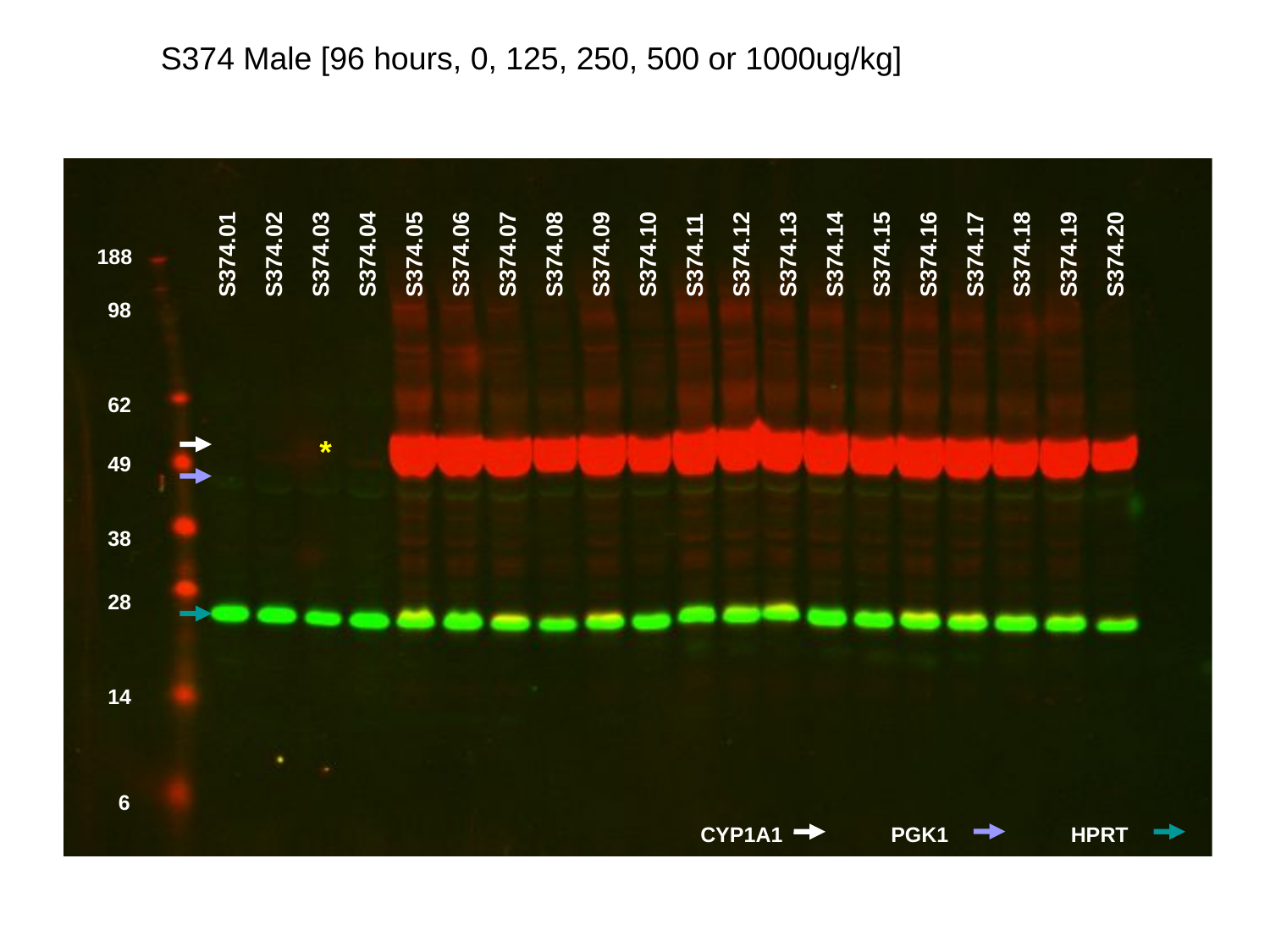

S374.01
S374.02
S374.03
S374.04
S374.05
S374.06
S374.07
S374.08
S374.09
S374.10
S374.11
S374.12
S374.13
S374.14
S374.15
S374.16
S374.17
S374.18
S374.19
S374.20
S374 Male [96 hours, 0, 125, 250, 500 or 1000ug/kg]
188
98
62
*
49
38
28
14
6
CYP1A1
PGK1
HPRT

## Slide 33
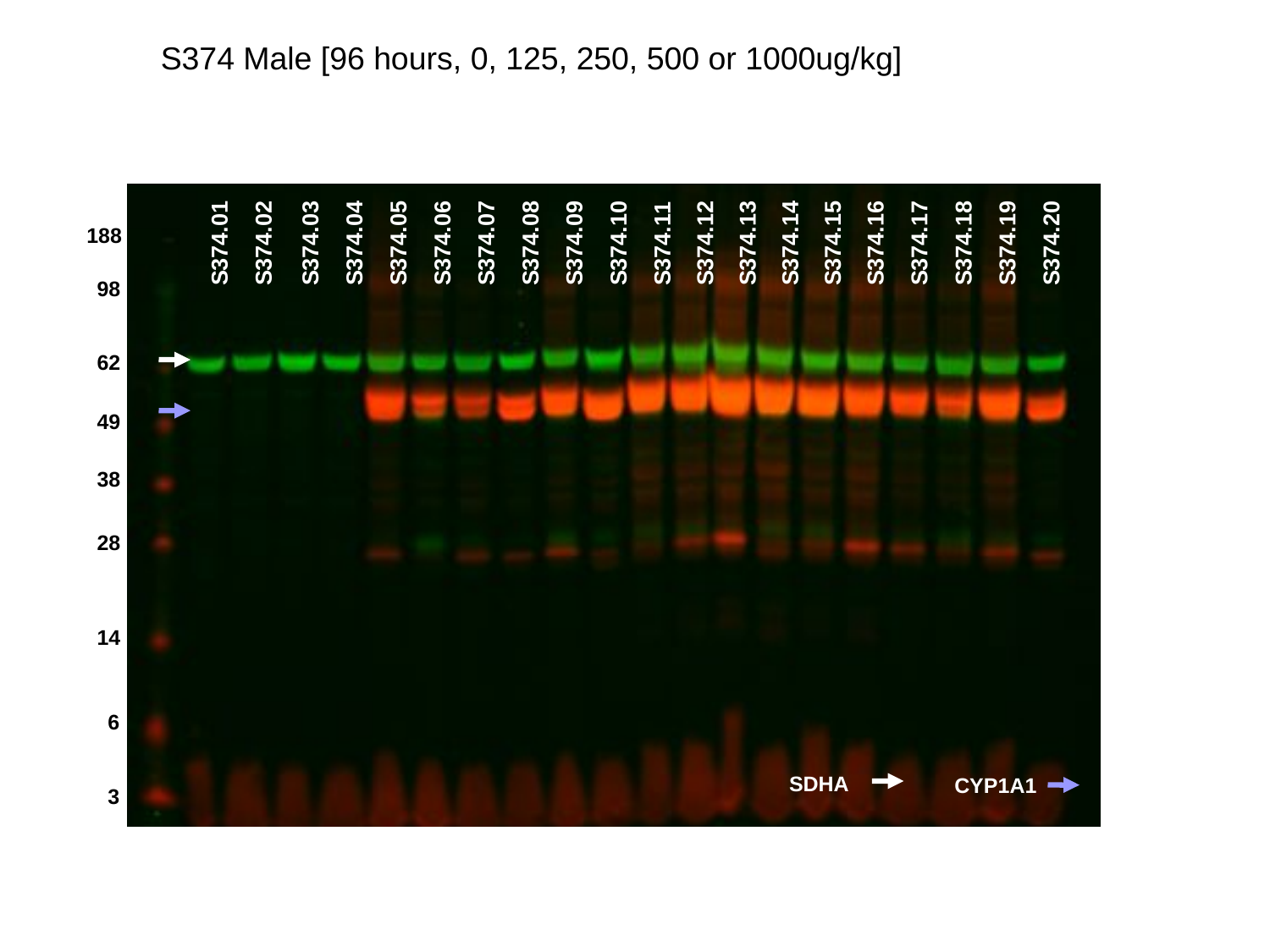

S374.01
S374.02
S374.03
S374.04
S374.05
S374.06
S374.07
S374.08
S374.09
S374.10
S374.11
S374.12
S374.13
S374.14
S374.15
S374.16
S374.17
S374.18
S374.19
S374.20
S374 Male [96 hours, 0, 125, 250, 500 or 1000ug/kg]
188
98
62
49
38
28
14
6
SDHA
CYP1A1
3

## Slide 34
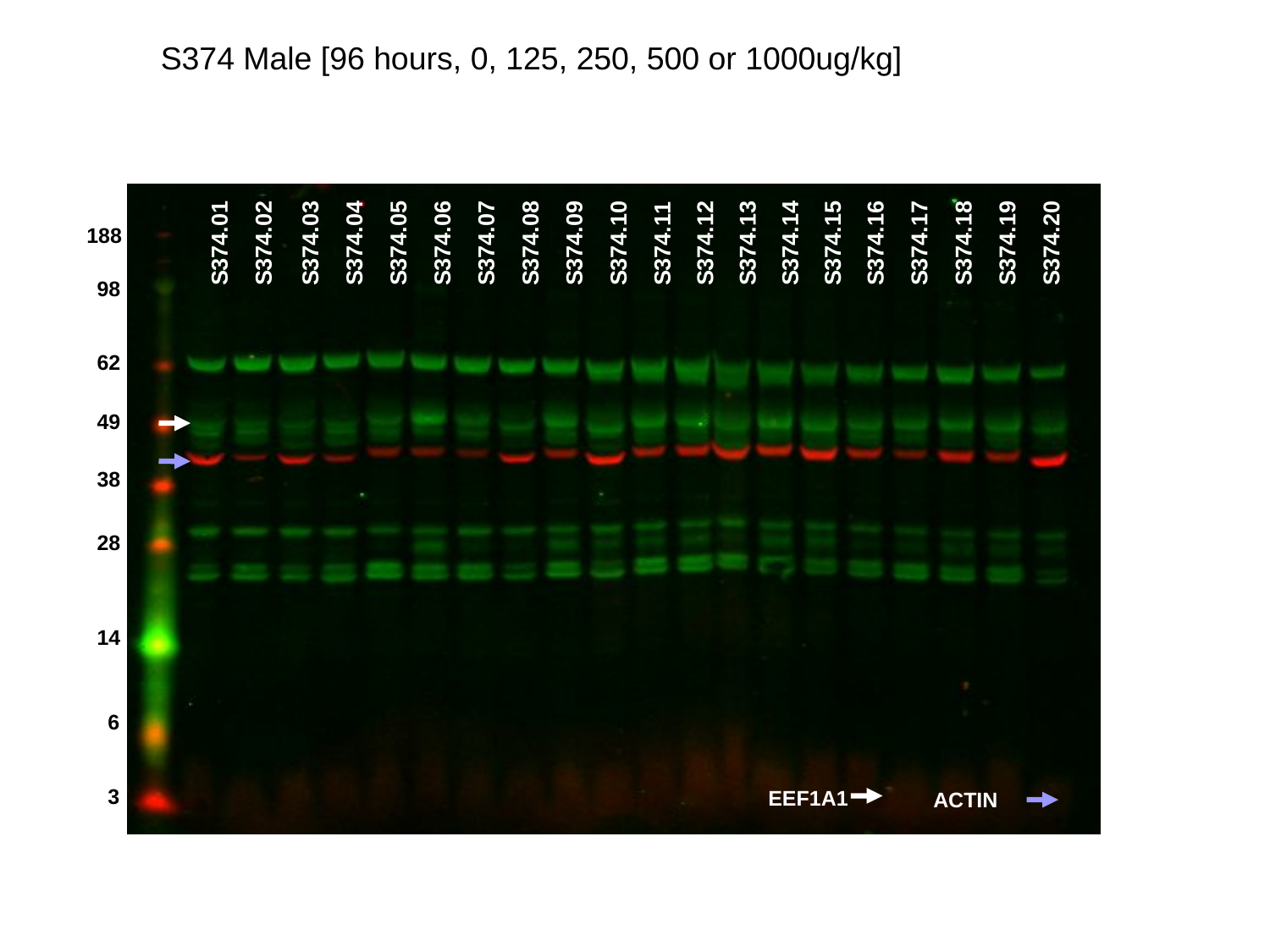

S374.01
S374.02
S374.03
S374.04
S374.05
S374.06
S374.07
S374.08
S374.09
S374.10
S374.11
S374.12
S374.13
S374.14
S374.15
S374.16
S374.17
S374.18
S374.19
S374.20
S374 Male [96 hours, 0, 125, 250, 500 or 1000ug/kg]
188
98
62
49
38
28
14
6
3
EEF1A1
ACTIN

## Slide 35
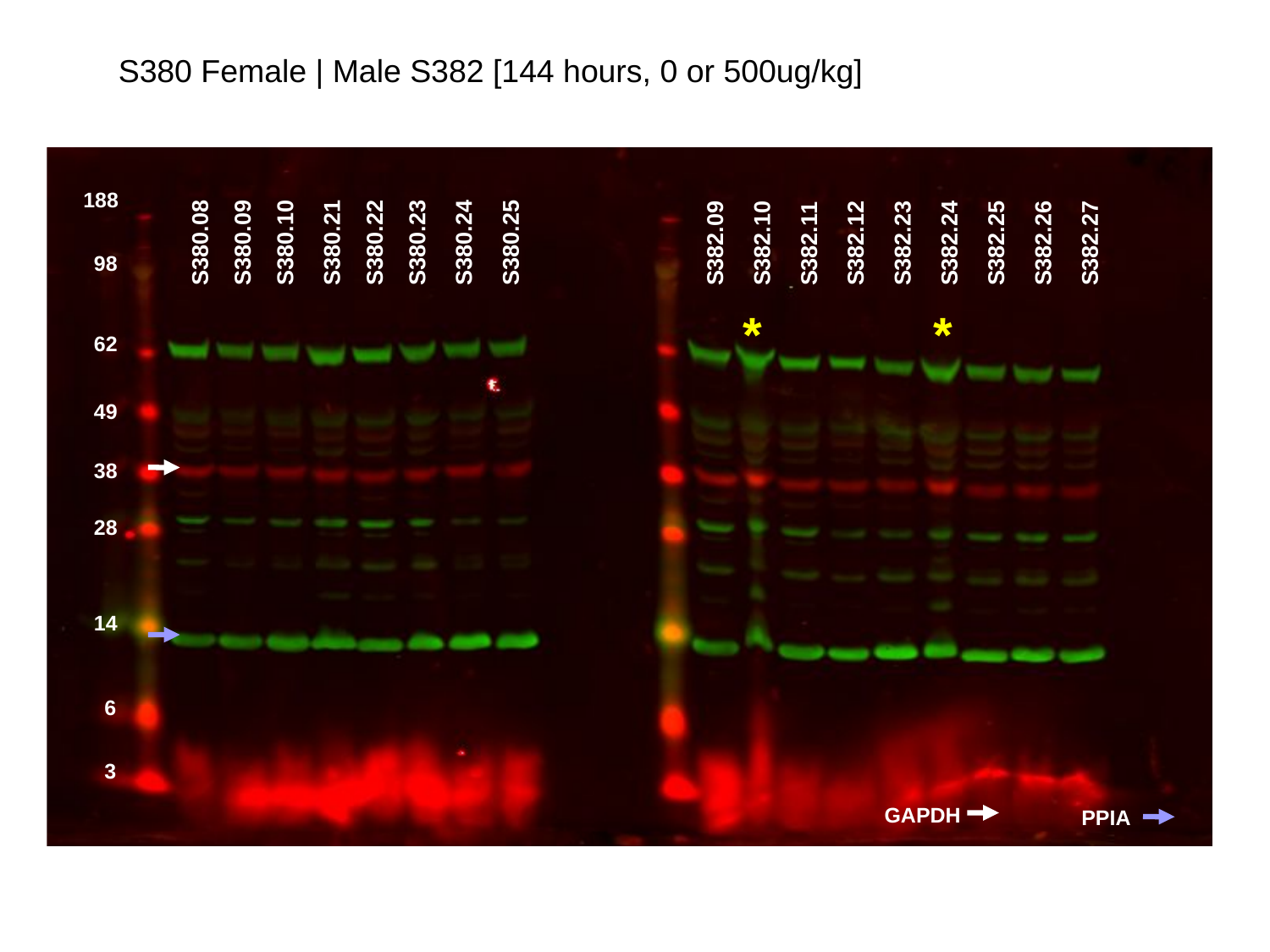

S382.09
S382.10
S382.11
S382.12
S382.23
S382.24
S382.25
S382.26
S382.27
S380.08
S380.09
S380.10
S380.21
S380.22
S380.23
S380.24
S380.25
S380 Female | Male S382 [144 hours, 0 or 500ug/kg]
188
98
*
*
62
49
38
28
14
6
3
GAPDH
PPIA

## Slide 36
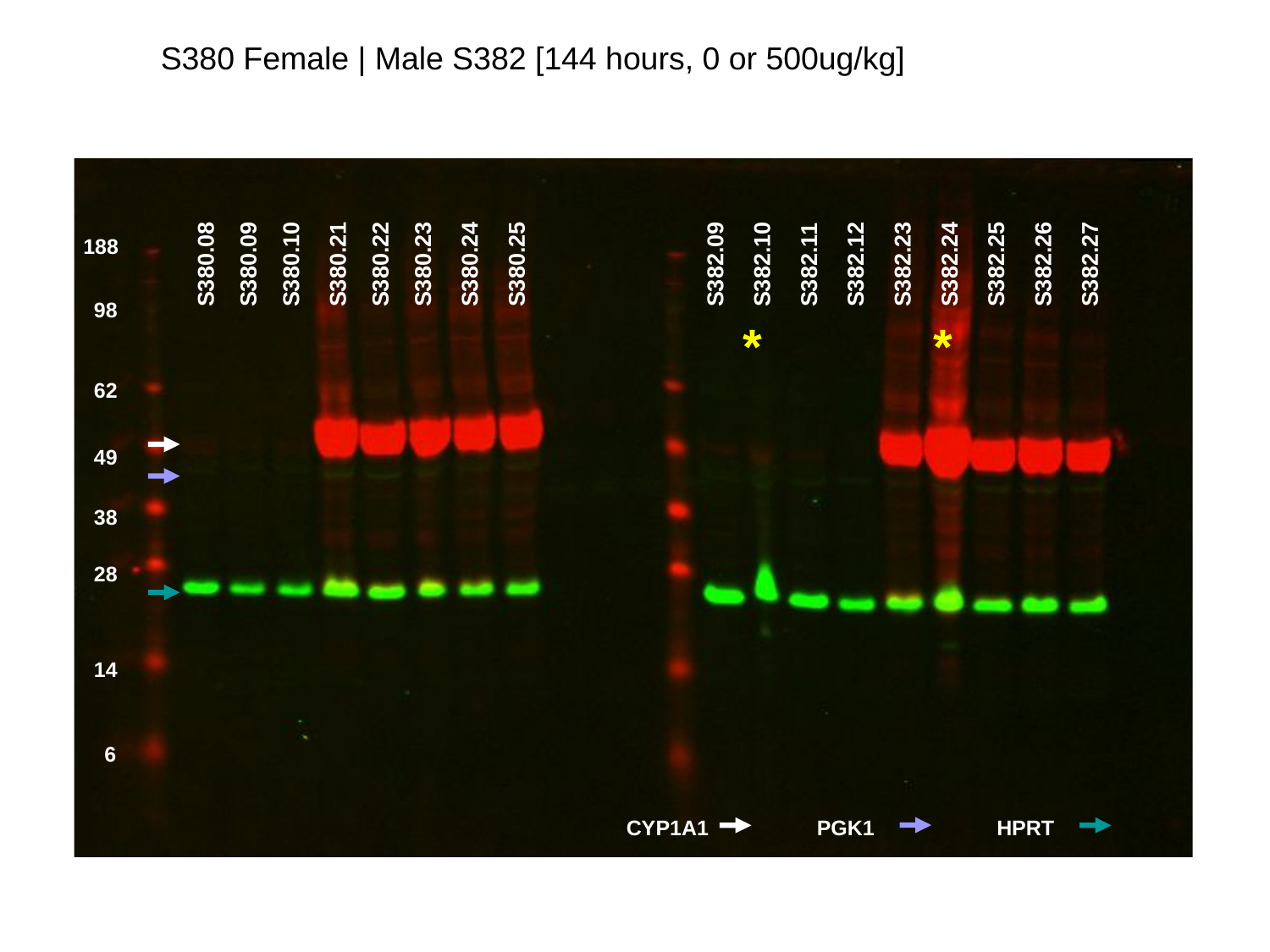

S382.09
S382.10
S382.11
S382.12
S382.23
S382.24
S382.25
S382.26
S382.27
S380 Female | Male S382 [144 hours, 0 or 500ug/kg]
S380.08
S380.09
S380.10
S380.21
S380.22
S380.23
S380.24
S380.25
188
98
*
*
62
49
38
28
14
6
CYP1A1
PGK1
HPRT

## Slide 37
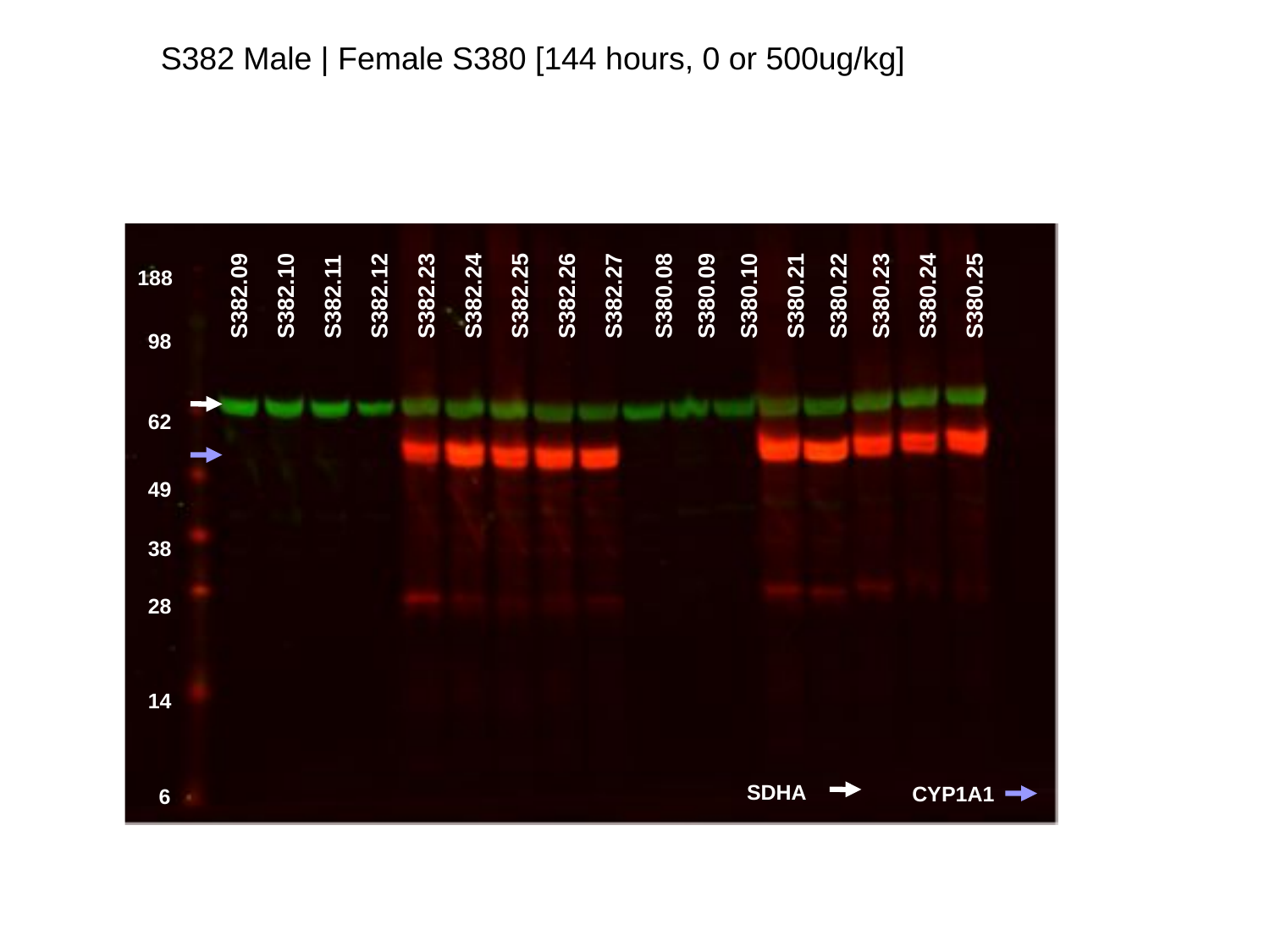

S382 Male | Female S380 [144 hours, 0 or 500ug/kg]
S382.09
S382.10
S382.11
S382.12
S382.23
S382.24
S382.25
S382.26
S382.27
S380.08
S380.09
S380.10
S380.21
S380.22
S380.23
S380.24
S380.25
188
98
62
49
38
28
14
SDHA
CYP1A1
6

## Slide 38
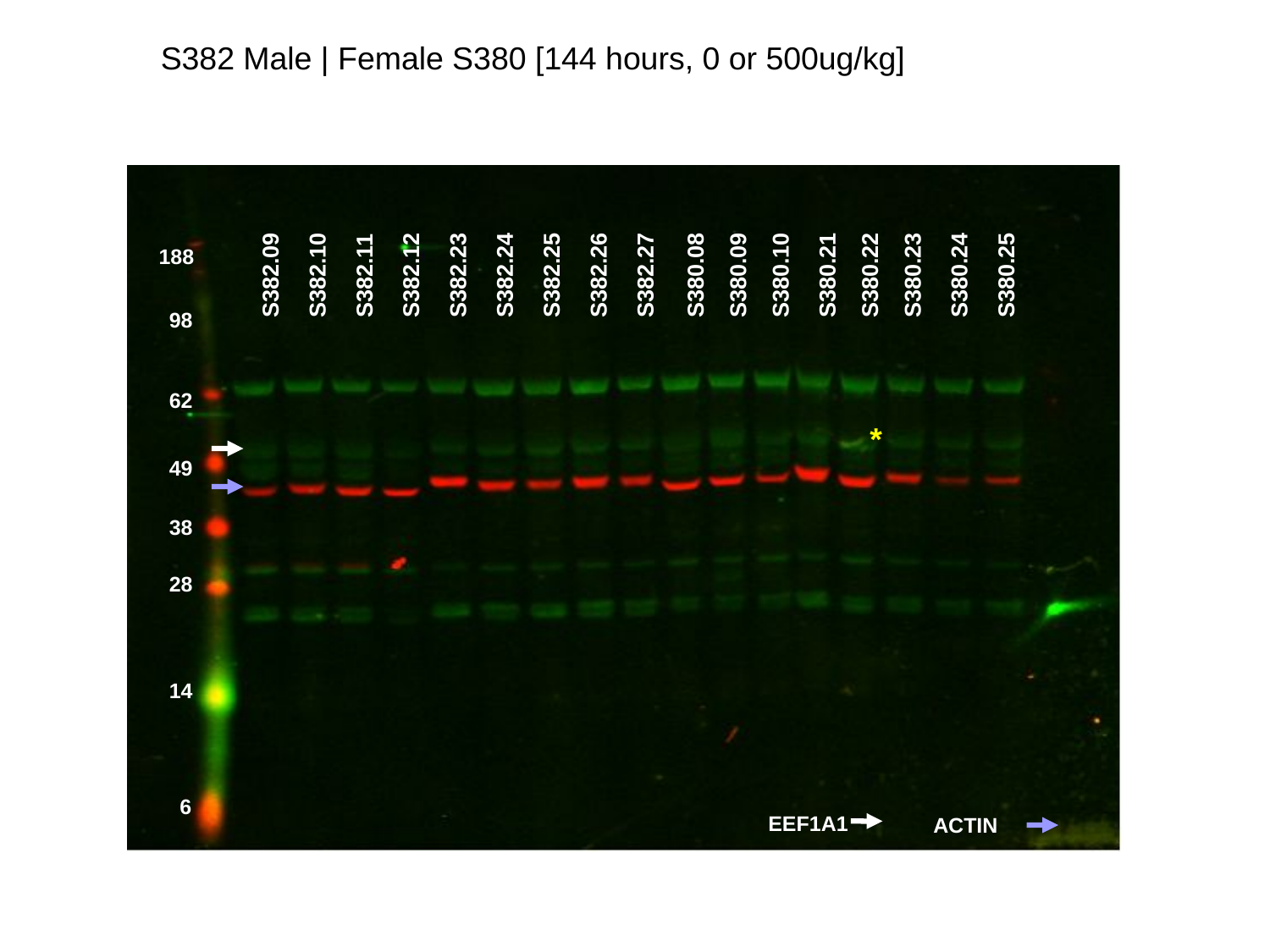

S382 Male | Female S380 [144 hours, 0 or 500ug/kg]
S382.09
S382.10
S382.11
S382.12
S382.23
S382.24
S382.25
S382.26
S382.27
S380.08
S380.09
S380.10
S380.21
S380.22
S380.23
S380.24
S380.25
188
98
62
*
49
38
28
14
6
EEF1A1
ACTIN
